# Tracking and optimizing toxic chemical exposure pathways through food trade: A case study in SCCPs contaminated seafood in China

**DOI:** 10.1093/pnasnexus/pgae205

**Published:** 2024-05-23

**Authors:** Shijie Song, Tao Huang, Yuting Xu, Zaili Ling, Ling Gou, Xiaoxuan Mao, Yuan Zhao, Kaijie Chen, Yao Liu, Zijian Wei, Jiaxin Wang, Hong Gao, Jianmin Ma

**Affiliations:** Key Laboratory for Environmental Pollution Prediction and Control, Gansu Province, Key Laboratory of Western China's Environmental Systems (Ministry of Education), College of Earth and Environmental Sciences, Lanzhou University, Lanzhou 730000, P. R. China; Key Laboratory for Environmental Pollution Prediction and Control, Gansu Province, Key Laboratory of Western China's Environmental Systems (Ministry of Education), College of Earth and Environmental Sciences, Lanzhou University, Lanzhou 730000, P. R. China; Key Laboratory for Environmental Pollution Prediction and Control, Gansu Province, Key Laboratory of Western China's Environmental Systems (Ministry of Education), College of Earth and Environmental Sciences, Lanzhou University, Lanzhou 730000, P. R. China; College of Agricultural and Forestry Economics & Management, Lanzhou University of Finance and Economics, Lanzhou 730101, P. R. China; Key Laboratory for Environmental Pollution Prediction and Control, Gansu Province, Key Laboratory of Western China's Environmental Systems (Ministry of Education), College of Earth and Environmental Sciences, Lanzhou University, Lanzhou 730000, P. R. China; Key Laboratory for Environmental Pollution Prediction and Control, Gansu Province, Key Laboratory of Western China's Environmental Systems (Ministry of Education), College of Earth and Environmental Sciences, Lanzhou University, Lanzhou 730000, P. R. China; Key Laboratory for Environmental Pollution Prediction and Control, Gansu Province, Key Laboratory of Western China's Environmental Systems (Ministry of Education), College of Earth and Environmental Sciences, Lanzhou University, Lanzhou 730000, P. R. China; Laboratory for Earth Surface Processes, College of Urban and Environmental Sciences, Peking University, Beijing, 100871, P. R. China; Key Laboratory for Environmental Pollution Prediction and Control, Gansu Province, Key Laboratory of Western China's Environmental Systems (Ministry of Education), College of Earth and Environmental Sciences, Lanzhou University, Lanzhou 730000, P. R. China; Key Laboratory for Environmental Pollution Prediction and Control, Gansu Province, Key Laboratory of Western China's Environmental Systems (Ministry of Education), College of Earth and Environmental Sciences, Lanzhou University, Lanzhou 730000, P. R. China; Key Laboratory for Environmental Pollution Prediction and Control, Gansu Province, Key Laboratory of Western China's Environmental Systems (Ministry of Education), College of Earth and Environmental Sciences, Lanzhou University, Lanzhou 730000, P. R. China; Key Laboratory for Environmental Pollution Prediction and Control, Gansu Province, Key Laboratory of Western China's Environmental Systems (Ministry of Education), College of Earth and Environmental Sciences, Lanzhou University, Lanzhou 730000, P. R. China; Laboratory for Earth Surface Processes, College of Urban and Environmental Sciences, Peking University, Beijing, 100871, P. R. China

**Keywords:** interprovincial food trade, China, seafood, short-chain chlorinated paraffins, risk assessment

## Abstract

Food safety is related to human health and sustainable development. International food trade poses food safety risks through the collateral transport of toxic chemicals that are detrimental to human health. Domestic interprovincial trade has similar effects within countries but has not been comprehensively investigated previously. Here, we assessed the effects of interprovincial trade on food safety and human dietary exposure to short-chain chlorinated paraffins (SCCPs), a group of emerging persistent toxic chemicals, in seafood across China by synthesizing data from field observation and various models. Our findings indicate that there is a higher level of SCCPs exposure risk in coastal provinces compared to inland provinces. Approximately, 70.3% of human exposure to SCCPs through seafood consumption in China was embodied in the interprovincial seafood trade in 2021. Specifically, the domestic trade led to a remarkable increase in SCCPs exposure in the coastal provinces in South China, attributable to low SCCPs pollution in these provinces and imported seafood from those provinces with high SCCPs pollution. In contrast, human exposure to SCCPs decreased in those coastal provinces in East China due to importing seafood from those provinces with low SCCPs concentrations. The interprovincial seafood trade routes were optimized by linear programming to minimize human exposure to SCCPs considering both shipping cost and health risk constraints. The optimized trade routes reduced the national per capita SCCPs exposure through seafood consumption by over 12%. This study highlights the importance of interprovincial food trade in the risk assessment of toxic chemicals.

**Significance Statement**

The rapid growth of food trade and logistics under globalization increases the human risk in those regions importing food from food supply regions contaminated by toxic chemicals. There is growing concern about how to reduce or avoid contaminated food embodied in food trade. Here, by synthesizing field observations and various models, we assessed the impacts of interprovincial seafood trade on human health via intaking contaminated seafood produced in different coastal seas across China, and propose a solution to reduce human exposure to toxic chemicals in seafood by optimizing interprovincial seafood trade. The results have significant implications for the regulations on food safety from food trade.

## Introduction

Food safety, a critical public health concern, significantly impacts the sustainable development and well-being of the global population (1–3). The increasing food demand due to the rapidly growing population and personal income has boosted the expansion of agriculture. However, this has triggered the use of various chemicals (4–6), such as pesticides, fertilizers, antibiotics, and persistent toxicants (7, 8) in agricultural activities. These chemicals enter the human food chain through the contaminated soil, water, and air (9, 10), posing significant threats to human health.

New dietary habits, regional and global food trade and logistics under globalization, and immigrants are new factors influencing the changes in the patterns of food production, distribution, and consumption in the past decades (11). These changes have challenged food safety and health. The food safety from a region can be transferred to other regions under this circumstance (12, 13). Liu et al. (14) demonstrated that the international rice trade increases human methylmercury exposure in Africa, Central Asia, and Europe. Undeman et al. (15) found that Inuit, a race in northern Canada, consuming imported fish with low polychlorinated biphenyl (PCB) pollution has reduced risk compared to the consumption of their traditional local marine mammals. It is imperative to ensure the safety of our food supply in a broader context of globalization and the rapidly growing food trade. The World Organization for Animal Health, International Plant Protection Convention, and the Codex Alimentarius Commission have established international standards for imports of animal, plant, and food commodities, respectively (16, 17). Some international agencies such as the United Nations Food and Agriculture Organization and the World Health Organization have been making collaborative efforts to safeguard public health and ensure adherence to the best practices in food trade (12). The food safety monitoring and tests in many countries are performed in a limited number of randomly selected samples, owing to the high cost (8). Many toxicants such as persistent organic pollutants (POPs) and mercury with a high cost of food monitoring have not received sufficient attention. These substances might pose long-term adverse health effects on humans (12).

The considerable scale and complexity of the food trade and supply system require a high cost for food safety inspection following the traditional method, owing to their reliance on extensive instrumentation and costly laboratory infrastructure (12). In contrast, numerical simulation offers a rapid, efficient, and alternative approach to assess food safety quantitatively (8, 13, 18). Huang et al. (13) have simulated the impact of the global fish trade on human POPs exposure by integrating a food trade pathway model and atmospheric transport model and reported that the global fish trade increased human exposure and associated risk to PCBs in areas with low ambient emissions of PCBs. Chen et al. (18) investigated the health risks from dioxin in the global pork trade and demonstrated that countries importing large amounts of pork meat from severely dioxin-contaminated Europe and the United States have an enhanced risk of dioxin exposure.

Ensuring national food safety and security heavily relies on the safety of interprovincial food trade within a country (19, 20). The absence of available statistics on food trade in most countries might explain the reason for neglecting the impact of interprovincial trade in previous studies (13, 20, 21). There is an urgent need for investigation in food safety associated with interprovincial and inter-regional food trade on a national and global scale, aiming to address existing research gaps and provide an overall assessment and recommendation for policymakers to formulate food safety policies.

China has the most extensive domestic food trade and logistics and has been the largest user of pesticides and fertilizers in the world (22). The trade and logistics of seafood, a vital food resource in China, is a key component in its food system. China has also been the largest producer and consumer of chlorinated paraffins (CPs) globally, with domestic production of CPs accounting for about 15% of global total CPs production in 2013 (23). Due to the environmental persistence, bioaccumulation, long-range transport potential, and toxicity (24), short-chain chlorinated paraffins (SCCPs) were included in Annex A of the Stockholm Convention on POPs (25) and the List of Priority Control New Pollutants (2023 version) in China (26). The present study focuses on the risk assessment of new and extensively used SCCPs (24, 27) in domestic seafood trade and logistics in China. We integrated the complex dynamics of interprovincial food trade into a comprehensive framework encompassing a (i) coupled marine food-web model, (ii) 3D-atmospheric transport model, and (iii) interprovincial food trade model. The logistic distance between food origins and destinations is a primary parameter in the food trade model from an economic perspective. However, the health risk due to toxic chemicals in traded food was not considered in previous studies. The present study made an effort to design an optimized seafood trade pathway by considering seafood transportation and health risks. This framework seeks to accurately quantify toxic chemical exposure originating from food consumption and trade. The scope of this study extends across the regional and nationwide context offering an extensive perspective on the implications of interprovincial food trade on toxic chemical exposure resulting from food consumption.

## Results

### SCCPs contamination in seafood

**Fig. 1.**
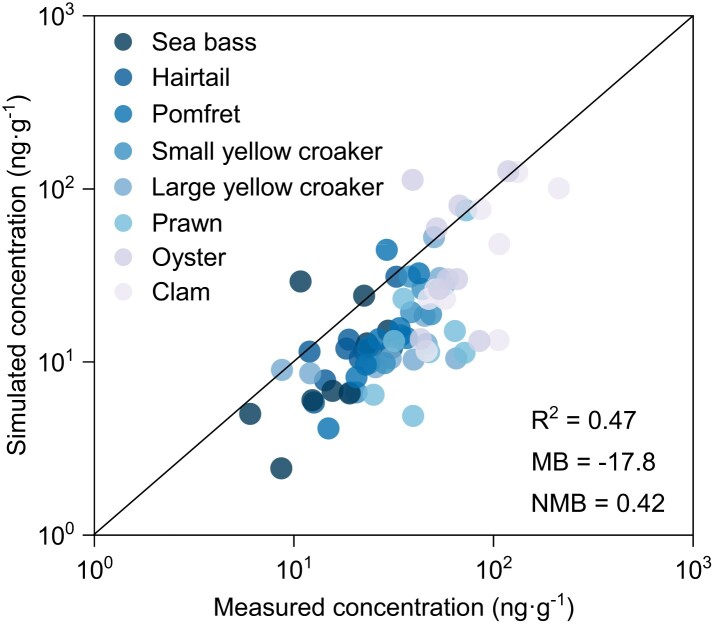
Comparison between simulated and measured SCCPs concentrations in seafood. Different species are highlighted by different colors. The solid black line indicates a 1:1 line. MB and normalized mean bias NMB are calculated as in Huang et al. (13).

The SCCPs concentrations in the eight marine species from the coastal waters (Fig. S1) in China were modeled using the marine food-web model (see Materials and methods section), and the results are illustrated in Fig. S2. The modeled concentrations agree well with the measured values (Fig. 1) with a statistically significant correlation between the modeled and measured concentrations (*R*^2^ = 0.47, *P* < 0.001). The results suggest that the marine food-web model can predict the accumulated SCCPs in the marine environment, although SCCPs concentrations in each species were slightly underestimated (Fig. S3). The model performance was evaluated by comparing modeled and measured SCCPs concentrations. 40.0 and 93.3% of the modeled SCCPs of eight aquatic organisms are within a factor of 2–5 of the measured SCCPs. The mean bias (MB) value under the 95% confidence interval (CI) of all species is −17.8 ng·g^−1^ wet weight (ww). Thus, the model could predict well SCCPs levels in the biotic and abiotic environments with reasonable accuracy. Our results also demonstrate the significant advantages of numerical modeling for studying SCCPs contamination levels in a wide range of marine species. Rather, the time consuming, expensive labor, and high financial cost would hinder comprehensive field observational studies of multipathways of SCCPs in the different environmental compartments over a large region and long period. In this sense, numerical modeling provides a useful approach to assess the environmental and health impacts of these contaminants on large temporal–spatial scales.

The modeled concentrations of SCCPs in major seafood from the coastal waters in China were associated with SCCPs levels in the marine environment (Fig. S4) and the primary diet composition of each marine species (Table S1) in the marine food web. Significant interspecies differences are observed in the SCCPs levels. The lowest SCCPs concentration is in pomfrets (mean of 5.3 ng·g^−1^ ww, 95% CI: 1.9–14.3), and the highest is in clams (23.7 ng·g^−1^ ww, 95% CI: 10.4–54.0). Clams, oysters, and prawns have higher SCCPs levels than other fish species. The SCCPs levels in large yellow croakers (11.3 ng·g^−1^ ww, 95% CI: 3.4–38.0), small yellow croakers (10.2 ng·g^−1^ ww, 95% CI: 4.3–24.3), and hairtails (7.3 ng·g^−1^ ww, 95% CI: 3.2–16.5) are higher than sea bass (5.4 ng·g^−1^ ww, 95% CI: 2.1–13.6) and pomfrets among the five fish species. Differences in habitats between marine fish and other marine species in the present study are a possible explanation for the observed interspecies variation. SCCPs are hydrophobic compounds, and the octanol–water partition coefficients (log *K*_OW_) of a series of commercial and synthesized SCCPs range from 4.01 to 8.67 (28), implying that they tend to be reserved by sediments. Prawns, clams, and oysters are exposed more frequently to the sediment than fish species (29), resulting in a higher body burden of SCCPs. Previous studies have reported high average concentrations of SCCPs of 940 ng·g^−1^ ww in bivalve matrices (30). The metabolic capability of SCCPs might influence the SCCPs body burden of marine species. Benthic species have a high accumulation capacity and a low elimination of heavy metals, organometallic compounds, and POPs (30, 31). However, the SCCPs metabolism in marine organisms is less known to date and requires more studies to validate the above results.

SCCPs levels in seafood were relatively higher in the nearshore water and decreased toward offshore (Fig. S2). The modeled higher SCCPs concentrations were identified in clams, large yellow croakers, sea bass, pomfrets, and small yellow croakers from the coastal areas of Shanghai, Shandong, and Zhejiang provinces. The highest SCCPs concentrations in hairtails, prawns, and oysters found in the coastal areas of Tianjin, Hebei, and Liaoning provinces, respectively. The mean SCCPs levels averaged over the eight marine species were higher in Shanghai and Tianjin than other coastal provinces, agreeing with higher SCCPs levels in the marine environments of the East China Sea than other coastal waters (Fig. S4). Eastern China is the most developed and industrialized region in China, where CPs have been used in electrical components, and plastic manufacturing and packaging (24), causing SCCPs pollutions. A gridded SCCPs emission inventory in China developed by Jiang et al. (23) reported the highest emission from 2008 to 2012 from the Shandong (742.3 tons), followed by Jiangsu (724.0 tons), and Zhejiang Provinces (573.4 tons), respectively. The accumulation of SCCPs in marine organisms is a critical process following SCCPs emission to the environment, which enhances human exposure through the ingestion of SCCPs-contaminated seafood.

### Estimated dietary intake of SCCPs in seafood

**Fig. 2.**
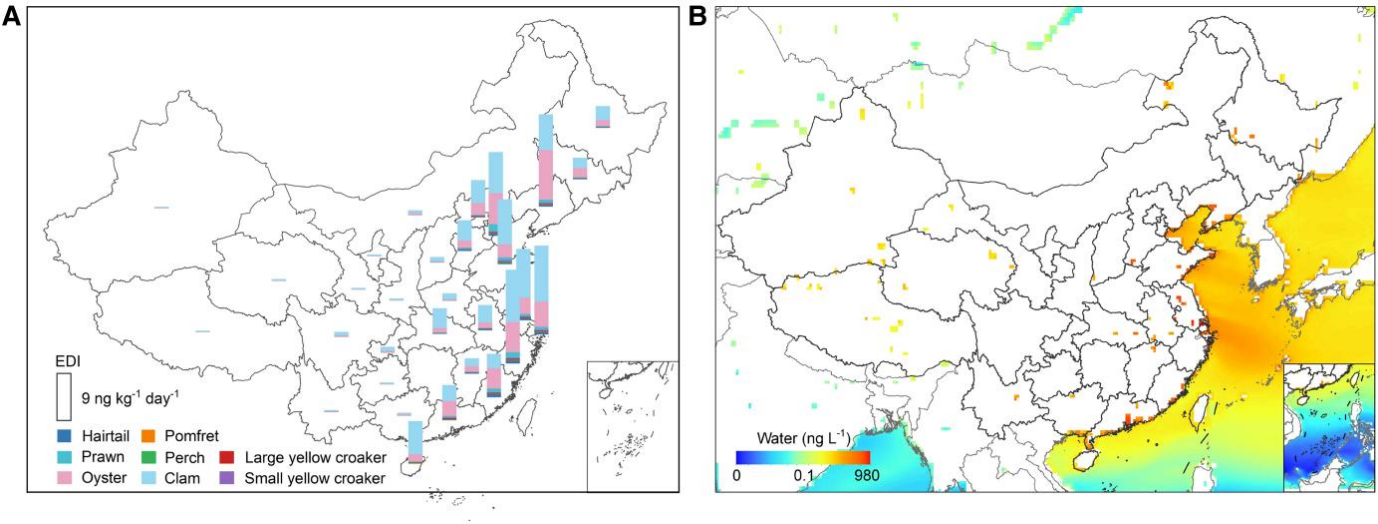
A) EDI of SCCPs for an adult consumer induced by seafood intake in 2021 and B) simulated annual average concentrations in water (ng·L^−1^) in 2021.

Seafood is a significant dietary source of human SCCPs exposure, especially in coastal provinces (Fig. 2). The average estimated daily intake (EDI) of SCCPs from seafood consumption is 5.8 ng·kg^−1^·day^−1^ (95% CI: 0.6–60.0) in China based on the consumption of seafood in different provinces (Fig. S5), well below the “no observed adverse effect level” of 10 mg·kg^−1^·day^−1^) proposed by European Food Safety Authority (32). The intake of clams among targeted marine species contributes 58.9% to the total seafood consumption (TSC) EDI, followed by oysters (30.3%), prawns (4.4%), hairtails (2.9%), large yellow croakers (1.4%), small yellow croakers (1.1%), pomfrets (0.7%), and sea bass (0.3%), respectively. Specific seafood consumption data are crucial to elucidate seafood intake as an exposure pathway. The per capita consumption of fish species in previous studies has been assumed to be equivalent to the aquatic products (33–35) due to the lack of species-specific databases in China. This hypothesis yields the average SCCPs EDI at 58.9 ng·kg^−1^·day^−1^ in China when the consumption frequency of each seafood is assumed to be equal to daily aquatic food consumption quantities (36). Thus, notable reductions in human exposure to SCCPs are observed on considering different seafood intake.

The SCCPs exposure through seafood consumption for fish consumers in different provinces is in the sequence of Zhejiang (17.7 ng·kg^−1^·day^−1^, 95% CI: 1.7–184.3) > Liaoning (17.3 ng·kg^−1^·day^−1^, 95% CI: 1.7–173.5) > Shanghai (16.7 ng·kg^−1^·day^−1^, 95% CI: 1.6–167.0) > Tianjin (15.7 ng·kg^−1^·day^−1^, 95% CI: 1.5–157.4). These are coastal provinces and cities with high seafood consumption rates of 50.4, 30.0, 52.9, and 33.8 g·day^−1^ in 2021 (Fig. S5), respectively. These values are 2.5-, 1.5-, 2.6-, and 1.6-fold of the mean seafood consumption rate in China. SCCPs exposure from eight seafood items in different provinces is shown in Fig. 2. Similar to the provincial seafood intake rates, the risk from SCCPs-contaminated hairtails in Zhejiang, pomfrets in Shanghai, large yellow croakers in Zhejiang and Shanghai, small yellow croakers in Shandong and Jiangsu, and sea bass in Shandong, Tianjin, and Jiangsu is relatively high. The mean SCCPs exposure from clams in China is 3.4 ng·kg^−1^·day^−1^ (95% CI: 0.3–35.7) with the highest exposure in Shanghai (10.5 ng·kg^−1^·day^−1^, 95% CI: 1.0–105.5). Liaoning province has the highest SCCPs exposure of 9.3 ng·kg^−1^·day^−1^ (95% CI: 0.9–93.3) from oyster consumption. The seafood contribution ratio indicates that calms are the main source of SCCPs exposure in Shanghai, Jiangsu, Zhejiang, Shandong, and Hainan. Apart from Guangdong and Liaoning, the contribution ratio of oysters ranks second in the remaining provinces.

### Provenance of seafood exposure to SCCPs across China

**Fig. 3.**
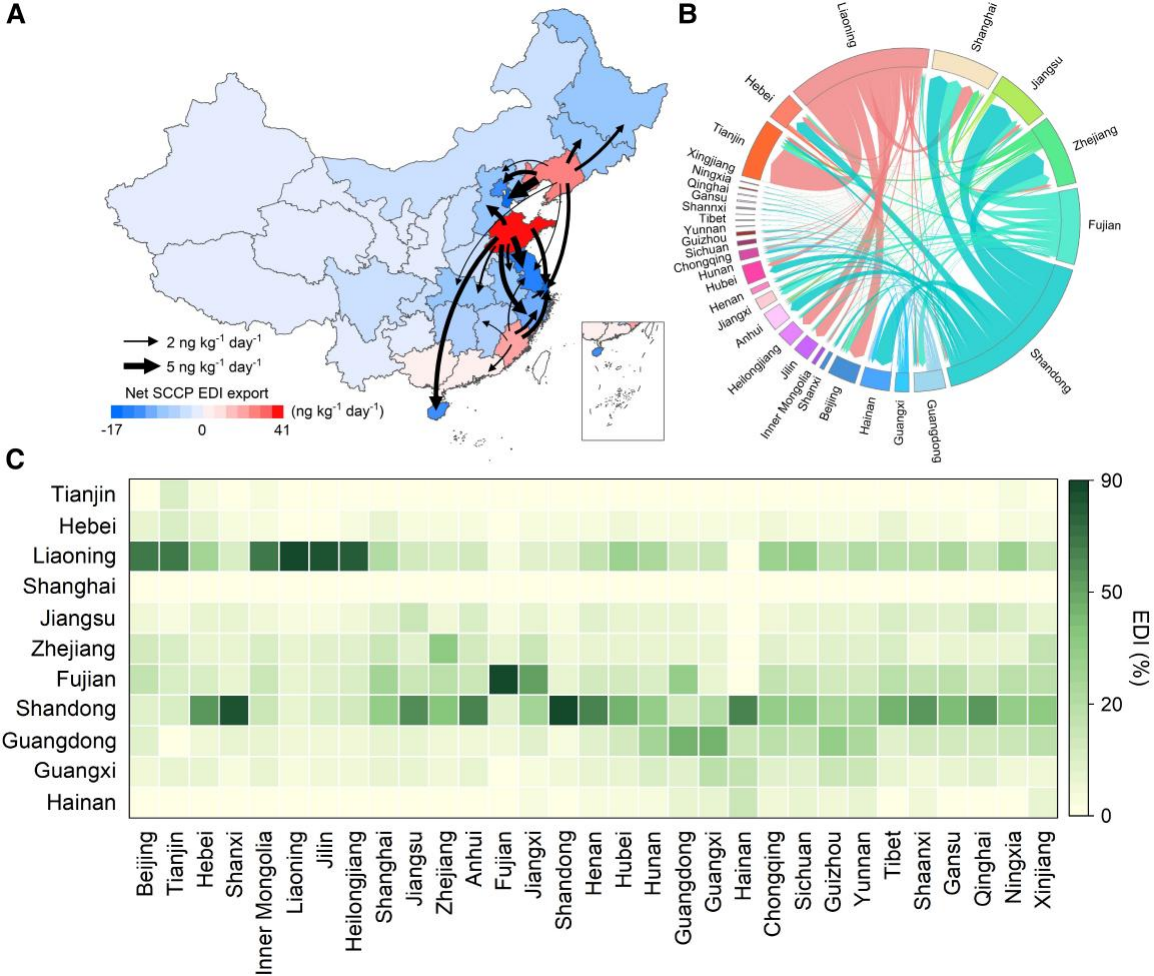
Transfer of EDI of SCCPs via interprovincial seafood trade across China in 2021. A) Net EDI of SCCPs export through interprovincial seafood trade and transfer of SCCPs EDI among 31 provinces. Positive value indicates regions of net EDI exports in the seafood trade, negative value indicates net EDI imports through interprovincial seafood trade. Arrow lines represent the pathway of EDI transfer embodied in the interprovincial seafood trade. B) Interprovincial EDI transfer embodied in the seafood trade is illustrated as a Circos plot. The width of each band represents the magnitude of EDI, and the band color represents the net inflow of EDI. The colors of the outer circular rings correspond to the provinces marked. C) Proportion of EDI (%) via locally produced and traded fish intake in each province. Each cell represents the EDI% from seafood consumption in each province indicated on the bottom x-axis to that produced in the region indicated by the left y-axis.

Trade results in a geospatial disconnection between the production and consumption of food products (13, 14) leading to the possibility of local consumers exposing to SCCPs via the dietary intake of contaminated food originating from external sea waters. A comprehensive analysis of the linkage between the geographical food origin and human health in food destinations embodied in food trade and logistics reveals another source–receptor relationship of SCCPs health exposure, apart from the source apportionment of SCCPs from their environmental transport. Figure 3A illustrates the SCCPs flow embedded in the interprovincial seafood trade and the associated contribution to the EDI of SCCPs in fish consumers across China. The consumers from Liaoning, Shandong, and Fujian, three coastal provinces, have >80% of SCCPs EDI by consuming local seafood. In contrast, the EDI of SCCPs in those inland consumers is via the consumption of seafood imported from coastal provinces near inland areas. Shandong, a significant seafood producer, exported the highest SCCPs to other provinces through the interprovincial seafood trade. Thus, the EDI of SCCPs in different provinces in 2021 is ranked as: Shandong 42.2 ng·kg^−1^·day^−1^ (95% CI: 4.0–447.9) > Liaoning (35.0 ng·kg^−1^·day^−1^, 95% CI: 3.5–351.4) > Fujian (18.4 ng·kg^−1^·day^−1^, 95% CI: 1.9–174.4) > Zhejiang (6.2 ng·kg^−1^·day^−1^, 95% CI: 0.6–62.7). The EDI embodied in fish trade between source (coastal) provinces and receiver provinces importing and consuming contaminated seafood from source provinces indicates that these four coastal provinces account for 82.8% of EDI. As a result, Shandong, Liaoning, Fujian, and Zhejiang as potential hot spots in the interprovincial seafood trade network from the perspective of trade-related health exposures play pivotal roles in bringing diet-borne SCCPs to other Chinese provinces.

The gravity model (see Materials and methods section) simulations identify that Shandong as the main fish or the EDI of SCCPs supplier to Jiangsu, Zhejiang, and Shanghai in 2021 contributed 60.5% (8.1 ng·kg^−1^·day^−1^, 95% CI: 0.8–86.2), 38.6% (6.8 ng·kg^−1^·day^−1^, 95% CI: 0.6–72.5), and 32.5% (5.4 ng·kg^−1^·day^−1^, 95% CI: 0.5–57.7) to the TSC SCCPs EDI in these three provinces, respectively. Liaoning as the second largest SCCPs EDI supplier contributed 10.8 ng·kg^−1^·day^−1^ (95% CI: 1.1–108.2) EDI to Tianjin, 4.7 ng·kg^−1^·day^−1^ (95% CI: 0.5–46.9) to Beijing, and 3.4 ng·kg^−1^·day^−1^ (95% CI: 0.3–34.3) to Jilin, respectively. Fujian contributed about 52.0% (1.5 ng·kg^−1^·day^−1^, 95% CI: 0.2–14.3), 31.2% (2.1 ng·kg^−1^·day^−1^, 95% CI: 0.2–19.5), and 26.8% (4.5 ng·kg^−1^·day^−1^, 95% CI: 0.5–42.4) of TSC SCCPs EDI to Jiangxi, Guangdong, and Shanghai via fish trade. Zhejiang contributed 11.2% (1.9 ng·kg^−1^·day^−1^, 95% CI: 0.2–19.1) of TSC SCCPs EDI to Shanghai. The intensity of the seafood trade flows provides a gross indication of the health risk of fish consumers. However, it is worth noting that the health risk from dietary exposure also depends on food consumption patterns (the process of seafood consumption).

Figure S6 provides the pathway of EDI transfer embodied in the seafood trade in China. The SCCPs intake from hairtail consumption was mainly from those imported from Zhejiang. Large yellow croaker consumed across China was imported from Fujian with >80% of SCCPs intake. 43.6% of SCCPs intake from the consumption of oyster species in China could be traced back to Fujian. Our investigation also suggests that 45.9 and 55.9% of the SCCPs exposure were attributed to the consumption of small yellow croakers and pomfrets from Zhejiang. Moreover, Shandong exported a high amount of SCCPs to other provinces via its prawn and clam trade, while Guangdong exports SCCPs to other provinces embedded in the sea bass trade.

### Impacts of interprovincial seafood trade on SCCPs exposure

**Fig. 4.**
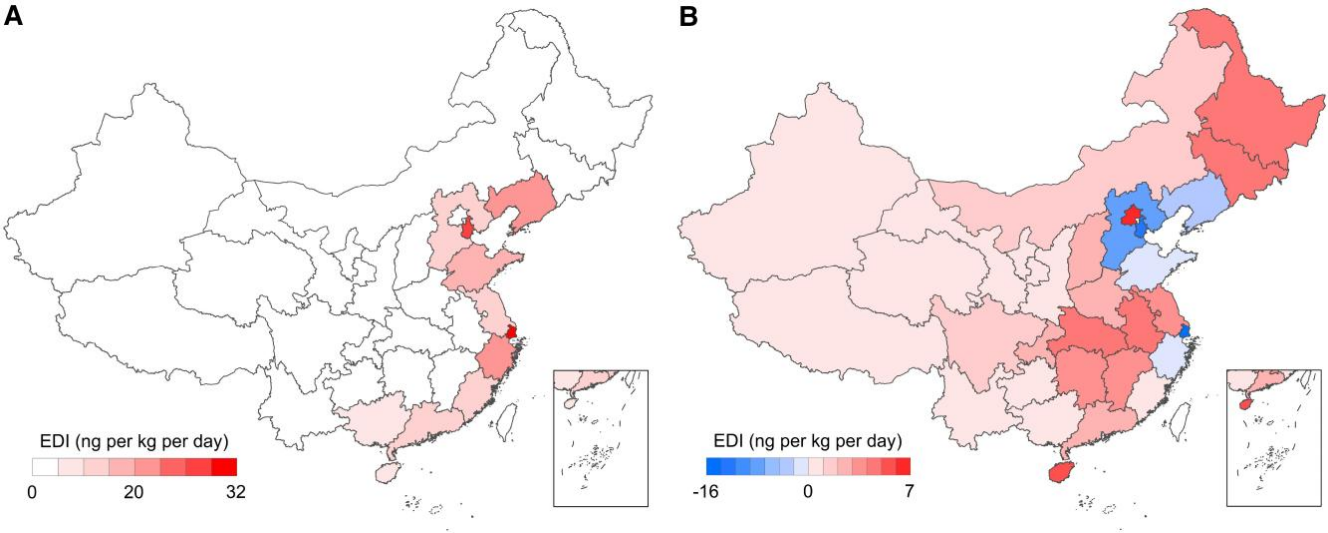
Dietary exposure of SCCPs was measured based on EDI via seafood consumption embodied by interprovincial seafood trade in 2021. A) EDI from no-trade simulation. B) Differences in EDI (EDI_DF_) between trade and no-trade model runs. The difference is estimated as EDI_DF_ = EDI_T_ − EDI_NT_, referred to as trade (EDI_T_) and no-trade (EDI_NT_) simulated EDI.

We set up a no-trade model scenario considering the absence of interprovincial seafood trade and a trade scenario including seafood trade. We compared the results between the two model scenarios to quantify the impacts of interprovincial seafood trade on SCCPs-related risk exposure in China. The no-trade simulation considers human exposure to SCCPs via local seafood consumption (Fig. 4A). Shanghai has the highest SCCPs EDI of 32.3 ng·kg^−1^·day^−1^ (95% CI: 3.1–335.3), followed by Tianjin (29.0 ng·kg^−1^·day^−1^, 95% CI: 2.8–298.3), Liaoning (19.7 ng·kg^−1^·day^−1^, 95% CI: 2.0–197.6), Zhejiang (17.8 ng·kg^−1^·day^−1^, 95% CI: 1.7–180.8), and Shandong (13.0 ng·kg^−1^·day^−1^, 95% CI: 1.2–138.0). All these coastal megacities and provinces are proximate to the major SCCPs land sources. The results of the EDI differences (EDI_DF_) between trade (EDI_T_) and no-trade scenarios (EDI_NT_) are illustrated in Fig. 4B. The EDI of SCCPs via seafood consumption in the interprovincial food trade scenario decreases by 70.0% (4.0 ng·kg^−1^·day^−1^), 14.0% (2.4 ng·kg^−1^·day^−1^), and 6.2% (0.8 ng·kg^−1^·day^−1^) in Hebei, Liaoning, and Shandong, respectively. Notably SCCPs EDI in Shanghai and Tianjin reduces by 93.5% (15.6 ng·kg^−1^·day^−1^) and 84.5% (13.3 ng·kg^−1^·day^−1^), respectively, attributed to seafood import from less contaminated coastal waters. In contrast, SCCPs EDI in Hainan, Guangxi, Jiangsu, Guangdong, and Fujian increased by 63.2% (5.0 ng·kg^−1^·day^−1^), 53.7% (0.3 ng·kg^−1^·day^−1^), 25.6% (3.4 ng·kg^−1^·day^−1^), 21.6% (1.4 ng·kg^−1^·day^−1^), and 3.1% (0.3 ng·kg^−1^·day^−1^), attributable to the consumption of seafood imported from highly SCCPs-contaminated coastal provinces. Similar results were reported by Huang et al. (13); the residents of low PCB-153 emission areas were at a higher pollution risk by consuming fish imported from high PCB-153 polluted areas. SCCPs EDI in Anhui, Hubei, and Jiangxi, the three inland provinces without marine fisheries, had been increasing. Although the EDI values in most inland regions are <1 ng·kg^−1^·day^−1^, the interprovincial food trade altered the health risk level of SCCPs in these regions via dietary intake. Liu et al. (19) examine the effects of farmed fish consumption and inter-regional trade on methylmercury exposure in China and highlights increasing risks in those regions importing fish from the marine environment with high methylmercury levels like East China. In contrast, our study focuses on SCCPs in the marine environments and explore how interprovincial trade modulates SCCPs exposure across different regions.

The impact of interprovincial food trade on human SCCPs exposure depends on seafood species (Figs. S8 and S9). The interprovincial food trade decreases to 6.0, 3.8, and 3.2 ng·kg^−1^·day^−1^ of SCCPs EDI due to oyster consumption in Hebei, Zhejiang, and Jiangsu, but increased to 2.1, 4.3, and 6.6 ng·kg^−1^·day^−1^ due to clam consumption, respectively. The mean SCCPs exposure in coastal provinces through seafood consumption embodied in interprovincial trade changed little. The interprovincial prawn trade especially reduced the mean EDI of SCCPs from 1.4 to 0.5 ng·kg^−1^·day^−1^ via prawn consumption in coastal provinces. The results suggest that optimizing seafood transaction and seafood supply chains between the regions become important to alleviate the SCCPs exposure, thereby reducing the nationwide exposure to SCCPs.

### Health risk from traded seafood

**Fig. 5.**
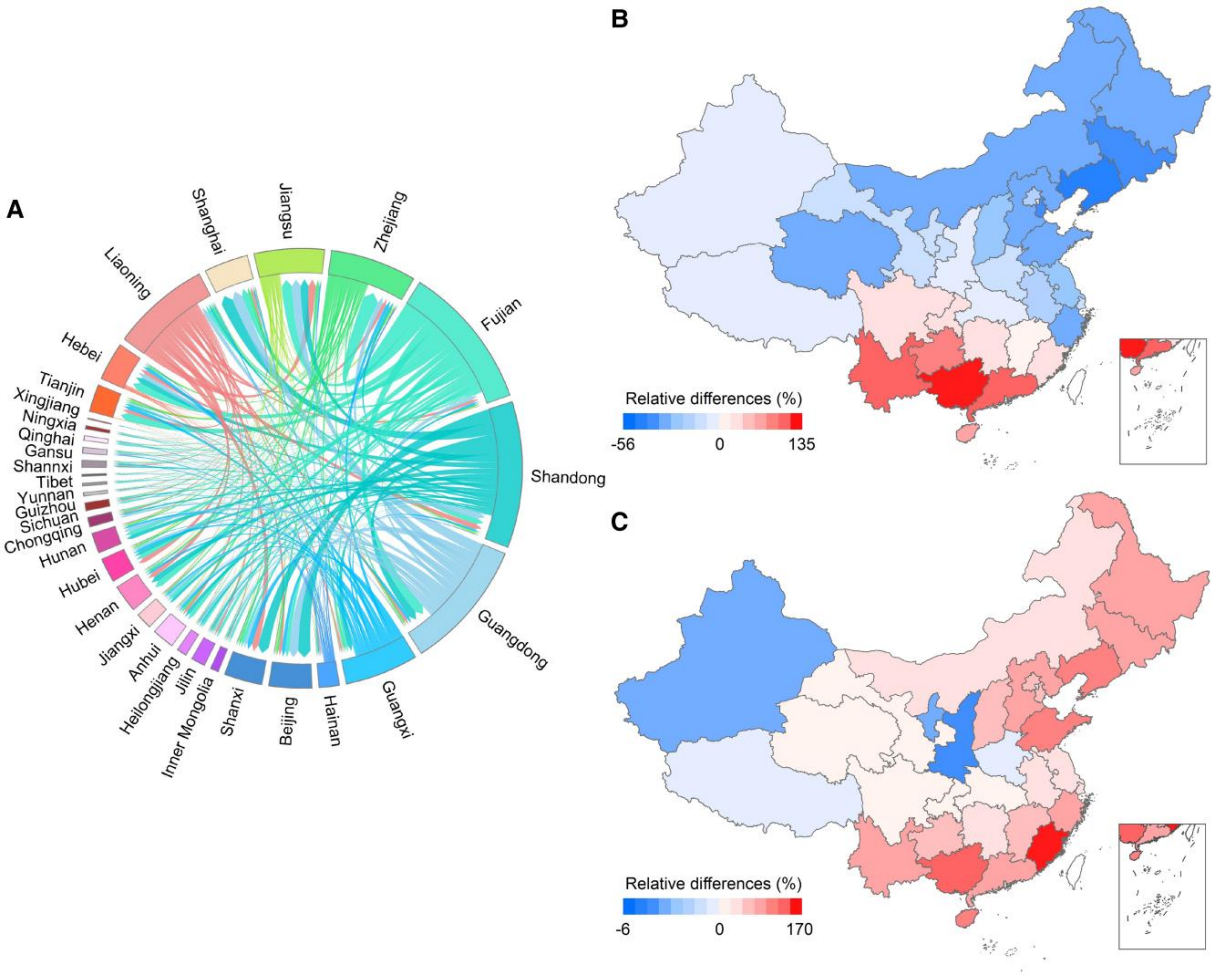
Optimized interprovincial seafood trade flows considering health risk impact on provincial EDI and cost from trade and logistics. A) Optimized interprovincial trade flows of seafood in 2021. B) Relative differences in provincial SCCPs EDI between optimized trade scenario considering health risk (EDI_optimized_) and current trade scenario without considering health risk (EDI_current_), calculated as EDI_RD_ = (EDI_optimized_ − EDI_current_) × 100/EDI_current_. C) Relative differences of the cost per unit trade seafood between optimized (Cost_optimized_) and baseline trade scenario (Cost_base_) estimated as Cost_DF_ = (Cost_optimized_ − Cost_base_) × 100/Cost_base_.

Fish trade and logistics in the present study are determined primarily by the gravity model (see Materials and methods section) that considers the transportation costs (shipping distance) and purchase amount only. Fish species caught in those seawaters with higher SCCPs contamination often yield higher risks, which, however, is neglected in the gravity model. To couple with the health effect, we further developed a multiobjective optimization model (see Materials and methods section) to optimize the interprovincial seafood trade flows considering both SCCPs EDI and the transportation cost simultaneously. Compared with the result from a baseline trade flow without including health risks (Fig. S10), we find that the optimized seafood trade flow reduced the mean EDI averaged over China by 12.8% (0.66 ng·kg^−1^·day^−1^) due to increasing traded seafood consumption from low SCCP-contaminated coastal seawaters, such as Fujian and Guangdong (Fig. 5). The highest EDI reduction occurred in Liaoning (55.7%), followed by Tianjin (49.7%), Jilin (48.7%), and Zhejiang (37.9%) (Fig. 5B). The fish consumers in these provinces should have taken in seafood from local coastal waters under heavier SCCPs pollution based on the Gravity modeling. The growing demand of healthy seafood in the seafood consumers from these provinces would the limit accessibility to SCCPs-contaminated seafood caught in nearby coastal waters in Eastern China but seek low SCCPs-contaminated fish caught in coastal waters in Southern China. In contrast, optimized seafood trade increases SCCPs exposure in Guangxi (134.9%), Guangdong (131.3%), Yunnan (125.1%), Guizhou (97.3%), Hainan (79.6%), etc., which are proximate to the coastal waters under lower SCCPs pollution in Southern China. Consequently, these provinces would be forced to increase their seafood import from high SCCPs-polluted regions to meet their market demands. The optimized trade flows for different seafood species and the impact on SCCPs EDI are shown in Figs. S11 and S12. The optimization modeling of seafood trade flows yields the largest decline of SCCPs EDI in oysters (58.2%), followed by clams (28.4%), prawns (7.7%), small yellow croakers (2.8%), and pomfrets (1.2%), respectively. The optimized trade pattern enhances 37.1% of transportation costs (0.8 billion Chinese yuan), particularly in Eastern and Southern China due to the seafood import from remote Southern China from a health perspective but reduces the EDI significantly. However, both trade and health costs increase in Southern and Southwestern provinces, such as Guangxi, Guangdong, Yunnan, Guizhou, Hainan, Fujian, and Sichuan.

## Discussion

This study assessed quantitatively the human SCCPs exposure via seafood consumption embodied in interprovincial seafood trade. The sampled marine species from the fishing grounds in Guangdong, Guangxi, and Hainan contain the lowest SCCPs levels due to long distances from the major SCCPs sources, thereby providing more safe seafood to consumers across China. In contrast, the fishing grounds in Shandong, Liaoning, and Fujian proximate to primary SCCPs sources are identified as potential hotspots of SCCPs-contaminated seafood.

The local or nearby fishing grounds often provide cheaper seafood to local and nearby seafood consumers due to the food availability and the lower transportation cost, which, however, is likely subject to a higher risk if the fishing grounds were more strongly contaminated than remote clean fishing grounds. We employed a linear programming to optimize interprovincial seafood trade routes, which considers both health and seafood transportation costs simultaneously. The approach optimizes seafood trade flow by implementing health effect in seafood logistics, resulting in increasing demands for seafood with low levels of SCCPs and decreasing demands for seafood with high SCCPs contamination. If the supply of seafood with low risks across China's coastal waters could not meet customers’ demands, we would compete for the seafood with low risks among provinces importing the seafood. In light of this, the optimized seafood trade flow might help designate seafood transportation routes with low cost and risk from a national perspective. As shown in Fig. 5, the optimized seafood trade flow reduced per capita SCCPs EDI by 12.8%, though enhancing the economic cost. If we could quantify the cost from reduced health risks, we would expect an overall reduction of the economic cost in the optimized seafood logistics.

The human dietary health risk embodied in the food trade is attributed to the consumption of locally produced food and food imported from other regions. Food safety is underpinned by the complexity of the nationwide food trade network (37) as it can promote the flow of SCCPs embedded in seafood among regions. Therefore, the spatial distribution of SCCPs-related health risks can be changed due to the interprovincial food trade. The dietary intake of SCCPs-contaminated seafood embedded in the food trade does not pose significant risks to seafood consumers in China based on the SCCPs risk criteria. However, concerns should be raised that seafood consumption in China has been increasing due to growing personal income, which enhances seafood consumption and corresponding risk to seafood consumers. The per capita consumption of cereals in China decreased by 23.4% from 2000 to 2021, while the per capita consumption of meat products such as aquatic products and pork rapidly increased by factors of 1.1 and 0.7, respectively (36). Although SCCPs emission has been decreasing in China (8), human SCCPs risk exposure might increase due to growing meat consumption through fishing industries and seafood trade. We always face the challenges in achieving sustainable and healthy seafood supplies on a national scale and highlight the significance of achieving a trade-off between ensuring food security and health benefit.

It should be noted that the present study focuses only on atmospheric pathways of SCCPs onto the marine environment. Since SCCPs are highly hydrophobic, the river runoff is likely another important pathway of SCCPs to the coastal waters. Our previous studies have demonstrated that atmospheric transport and deposition dominate toxic chemicals entering into China's marine environment and the riverine discharge via terrestrial runoff plays almost a negligible role (33, 38) and hence is not considered here. There are other limitations in the present study (i) The intake level of seafood consumers of different age groups was not considered in this study, which could affect seafood consumption and trade (14, 39). The national health survey data should be implemented into our model framework to improve the risk assessments and accurately evaluate individual intake differences and health risks. (ii) This study only evaluated the risk of human exposure to SCCPs in seafood. The assessments can be extended to other food items, typically meat products where organic chemicals are easily accumulated. (iii) Our investigation focused on SCCPs as a representative food contaminant. It is worth noting that the methodology presented in this study has the potential to be applied to other hazardous substances, including but not limited to mercury, perfluorinated compounds, and flame retardants such as polybrominated diphenyl ethers.

## Materials and methods

### Study framework

The present study conducted case studies to assess the impact of interprovincial trade on human exposure to SCCPs via the consumption of SCCPs-contaminated seafood. We collected the seafood samples from the main fishing grounds across China's coastal waters and examined the SCCPs levels. We investigated the spatial distribution of SCCPs in the atmosphere, soil, seawater, and sediment in China by using a modified version of the Canadian Model for Environmental Transport of Organochlorine Pesticides (CanMETOP) model and a high-resolution SCCPs emission inventory (13, 40). We calculated the SCCPs concentration in seafood considering the intricate relationship of predator–prey interactions using a marine food-web model. The fish transportation from fishing grounds (origins) to the destination was quantified using the food trade model. The observed and simulated levels of SCCPs in seafood, SCCPs sources, and the consumption patterns of fish consumers across different provinces were integrated to assess the risk exposure to SCCPs and the impact of seafood trade on fish consumers in China.

### Sampling and analysis

The sampling map and sites are shown in Fig. S13. Samples were collected in 2021 from 10 fishing grounds across China's coastal water. Eight species of seafood were selected: hairtail (*Trichiurus lepturus*), large yellow croaker (*Larimichthys crocea*), small yellow croaker (*Larimichthys polyactis*), sea bass (*Lateolabrax japonicus*), pomfret (*Pampus argenteus*), prawn (*Penaeus chinensis*), clam *(Mactra chinensis*), and oyster (*Crassostrea gigas*). All marine species samples were pools of five individuals. Table S2 shows scientific classification and dietary characteristics of marine species in this study. Detailed sample preparation and analysis methods are referred to the Supplementary Section S1 and Fig. S14.

### Atmospheric transport model

A modified version of the CanMETOP model was used in this study to model the spatiotemporal variations of SCCPs in the atmosphere, soil, seawater, and sediment (13). CanMETOP is a 3D-atmospheric transport model coupled with a dynamic fugacity-based soil–air exchange model and water–air exchange model based on the two thin film theory (40). The model simulates atmospheric transport, deposition, and the multiphase exchange of POPs between different environmental compartments (13, 41–43). The recently developed model has improved the water–sediment–phytoplankton exchange modules in CanMETOP (44). Phytoplankton resides at the bottom tropic level of the marine food web in marine ecosystems and plays a key role in transferring organic chemicals from water to fish (45). The water–sediment–phytoplankton modules have been updated in CanMETOP to simulate POPs concentrations in the marine ecosystem (13, 44). The model domain covered the entire China and adjacent oceans with a 0.25° longitude by 0.25° latitude horizontal resolution. Model input data include meteorological parameters, land cover, the historical data of atmospheric SCCPs emission inventory, and the physicochemical properties of SCCPs. The detailed model configuration used in this study can be found in Refs (8, 44). In this study, the model was integrated from 2008 to 2021 considering the accumulation of SCCPs in the Chinese marine environment. Further details and validation of CanMETOP modeling results model are provided in Section S2 and Fig. S15.

### Marine food-web model

There are different organisms at various trophic levels in the marine ecosystem, and the predation relationship among them is complex (13, 44). Zooplankton and benthic invertebrates are the main prey of the high trophic level fish (46). Fish consume algae, phytoplankton, animal and plant residues, fine particles in water, and sediments as food (47). Further details are referred to the Sections S3 and S4.

### Gravity model for interprovincial seafood trade

China's seafood production (13,742.9 kilotons) was higher than its consumption (36), indicating that the interprovincial seafood flow could meet the seafood demand from various regions of China. Therefore, the self-sufficiency of seafood and food security was ensured in China.

In this study, we adopted a well-known gravity model developed by Leontief and Strout (48) to predict interprovincial seafood trade flows. The interprovincial trade flows in the standard Leontief–Strout gravity model are specified as a function of the total regional outflows, inflows, and transfer cost, which is usually proxied by a distance function (49, 50). The gravity model is used extensively to evaluate the inter-regional commodity flows (50–53) and estimate the impact of inter-regional seafood trade on food safety (54, 55). The mathematical simplicity and intuitive nature of the gravity model and the reasonability of its empirical results grant its popularity and success in calibrating trade flows (56). The comparative assessment of Sargento (57) with other alternative models also indicated that the gravity model was well suited to explain trade flow behavior. The total supply and demand of seafood from each province in gravity modeling were used to represent their mass (Fig. S16), and the distance between provincial capital cities represented the distance between provinces. Detailed descriptions of the gravity model and model-predicted interprovincial seafood trade flow are presented in Section S5 and Figs. S10 and S17.

### Health exposure assessment

The EDI (g·kg^−1^·day^−1^) of SCCPs via seafood consumption can be calculated by:


(1) $$\mathrm{ED}I_{i}=\frac{C_{i}\times I_{i}}{W},$$

where *C_i_* is the SCCPs (ng·g^−1^) concentration in seafood species *i*, *I_i_* is the daily per capita consumption of the *i*th seafood (g·day^−1^), and *W* is the standard body weight of a Chinese male adult (63 kg) (58). The daily consumption of hairtail, large yellow croaker, small yellow croaker, sea bass, pomfret, prawns, clams, and oysters in each province in 2021 was estimated by the ratio of the market size and population of each province in mainland China in the same year (20, 36), as shown in Fig. S5. We also compared the reported daily seafood consumption and explored the deviations (Section S6 and Fig. S18). The TSC-induced EDI of SCCPs is the sum of EDI*_i_* of the eight seafood species.

### Optimization of interprovincial seafood trade routes

The optimization procedure for traded seafood is developed by using the Matlab optimization toolbox based on multiobjective linear programming (59–62). The gravity model output was used as the initial guess. The cost and risk were weighed separately. We adopted a multiobjective optimization approach to calculate the optimal trade route that minimized the cost from seafood logistics and health risk. Details are described in Section S7.

## Supplementary Material

pgae205_Supplementary_Data

## Data Availability

Gridded fish catch data are available on the Sea Around Us at http://www.seaaroundus.org. Gridded SCCPs emission inventory is publicly available at http://kleppc.lzu.edu.cn/contents/80/192.html. The other data supporting the findings of this study are available within the article and its Supplementary Information. The model codes are available at https://doi.org/10.5281/zenodo.11214066.
