# Supplementary material for: Tracking and optimizing toxic chemical exposure pathways through food trade: A case study in SCCPs contaminated seafood in China
Source: PNAS Nexus. 2024 May 23;3(6):pgae205. doi: 10.1093/pnasnexus/pgae205 (PMC11154648; doi:10.1093/pnasnexus/pgae205)
Supplement: pgae205_Supplementary_Data [file pgae205_supplementary_data.zip › PNASNEXUS-PNASNEXUS-2024-00402R-s01.docx]

**
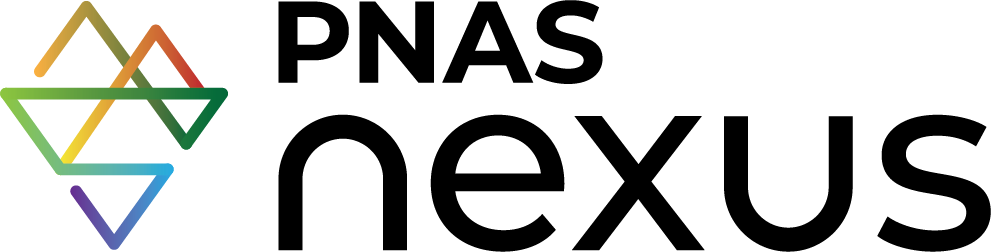
**

**Supplementary Information for**

Tracking and optimizing toxic chemical exposure pathways through food trade: a case study in SCCPs contaminated seafood in China

Shijie Song^a^, Tao Huang^a,^*, Yuting Xu^a^, Zaili Ling^b^, Ling Gou^a^, Xiaoxuan Mao^a^, Yuan Zhao^a^, Kaijie Chen^c^, Yao Liu^a^, Zijian Wei^a^, Jiaxin Wang^a^, Hong Gao^a^, Jianmin Ma^c^

^a^ Key Laboratory for Environmental Pollution Prediction and Control, Gansu Province, Key Laboratory of Western China’s Environmental Systems (Ministry of Education), College of Earth and Environmental Sciences, Lanzhou University, Lanzhou 730000, P. R. China

^b^ College of Agricultural and Forestry Economics & Management, Lanzhou University of Finance and Economics, Lanzhou 730101, P. R. China

^c^ Laboratory for Earth Surface Processes, College of Urban and Environmental Sciences, Peking University, Beijing, 100871, P. R. China

*Corresponding Author: Tao Huang

Email: [huangt@lzu.edu.cn](mailto:huangt@lzu.edu.cn)

**This PDF file includes:**

Supplementary text

Figures S1 to S19

Tables S1 to S11

SI References

**Other supplementary materials for this manuscript include the following:**

Datasets S1

**Supplementary Information Text**

**1. Sample analysis**

**Materials and reagents.** Commercial standards of SCCPs mixtures (C_10-13_, 51.5%, 55.5% and 63% chlorination, 100 μg·mL^-1^ solutions in cyclohexane) were purchased from Dr. Ehrenstorfer GmbH (Augsburg, Germany). Internal standard of *ε*-hexachlorocyclohexane (*ε*-HCH, 10 ng·μL^-1^) in cyclohexane and surrogate standard of ^13^C_10_-trans-chlordane (100 μg·mL^-1^) in n-nonane were obtained from Cambridge Isotope Laboratories (Andover, MA). Dichloromethane and n-hexane with high performance liquid chromatography grade were purchased from Fisher Scientific, and Silica gel (0.063-0.100 mm particle diameter) were from Sigma-Aldrich. Florisil (60-100 mesh) was bought from J&K Scientific. Anhydrous sodium sulfate and concentrated sulfuric acid were from Sinopharm Chemical Reagent Beijing Co., Ltd. Prior. Moreover, florisil was baked at 140°C overnight, silica gel was activated at 550 °C for 12 hours, and anhydrous sodium sulfate was baked at 660 °C for 6 hours prior to use. The preparation of 44 % acid silica gel was prepared by mixing 200 g activated silica gel and 157 g concentrated sulfuric acid, and stored in desiccator.

**Sample extraction and cleanup.** The samples were extracted and purified according to a previously published method^1^. In brief, 2.0 g of freeze-dried and homogenized fish sample was spiked with 2 ng of ^13^C_10_-trans-chlordane and extracted by a mixture of dichloromethane and n-hexane (1:1, v/v) on Dionex ASE350 accelerated solvent extractor (Thermo Fisher Scientific, Sindelfingen, Germany). The extraction program was 5 min heating, 10 min static extraction, three cycles at 100°C and 1500psi, 70% flush volume, and 60 s N_2_ purge time. Then the extract was concentrated and further exchanged to n-hexane (10 mL). Afterwards, 1mL of the extract was taken for measurement of lipid content by gravimetric analysis. The other 9 mL extract was transferred into a multilayered column which was packed with 3 g of florisil (3% water deactivated), 2 g of neutral silica gel (3 % water deactivated), 5 g of acidified silica gel (44% sulfuric acid), and 3.5 g of anhydrous sodium sulfate from bottom to top. The column was pre-cleaned with 50mL hexane before the extract was applied. After the extract had been applied, the column was eluted with 40mL of hexane and 100mL of a 1:1 v/v mixture of n-hexane and dichloromethane. The cleaned extract was evaporated almost to dryness, then 2.5 ng of ε-hexachlorocyclohexane was added to act as an internal standard and the sample was diluted to 200μL.

**Instrumental analysis and SCCPs quantification.** Identification and quantification of SCCP were performed using a gas chromatograph coupled with an electron-capture negative-ionization mass spectrometer (Thermo Fisher Trace 1300 GC-ISQ LT), according to the method reported previously^1^. 1 μL of sample was injected using auto sampler in splitless mode. The separation column used was a DB-5 MS capillary column (30 m length, 0.25 mm i.d., 0.25 μm film thickness). Methane was used as the reagent gas in a rate of 1 mL·min^−1^. Additionally, helium was used as the carrier gas at a rate of 1 mL·min^−1^. The oven temperature was initially isothermal 120 °C for 1 min, further increased to 280 °C at a rate of 15 °C·min^-1^, held for 15 min. The temperatures of the injector, interface and ion sources were set to 280 °C, 280 °C and 200 °C, respectively. The two most abundant [M-Cl]^-^ isotopes have been monitored in the selected ion monitoring (SIM) mode for quantification and confirmation of SCCP congeners^2^.

The quantification of sampled SCCP were performed using the following equations^2,3^:

$Total Corrected Relative Area=\sum\frac{Area \left( i \right)}{Area \left( istd \right)\times chlorine Atom \left( n \right)\times Abundance}$ (S1)

$Total Cl\%=\sum\left[ Cl\% \left( i, caculated \right)\times\frac{Corrected Relative Area \left( i \right)}{Total corrected relative area} \right]$ (S2)

$Total responce factor=m \times Total Cl\%+n$ (S3)

where *i* is the specific SCCPs homologue, Cl % represents the degree of chlorination in the SCCP homologue, and m and n are the slope and intercept of the linearity relationship of chlorination and total response factor, respectively.

The chlorine content of SCCPs in the sample can be calculated by eq S4. The total response factor of SCCPs in the sample can be determined from the chlorine content using the linear correlation in Fig. S14. It should be noted SCCPs quantification in this study was performed using the ECNI-MS method established by Reth et al^2^. Since the ECNI-MS method often failed in congener groups with less than 5 chlorine atoms, relative abundance of higher chlorinated SCCPs might be overestimated and contribute more to the detected chlorine contents. As a result, the differences of chlorine contents between the manufacturer declaration and our calculated value occurred. Such differences also took place in other studies. Once the total response factor was determined, the total SCCPs amount in the sample can be calculated as follows.

$CPs amount=\frac{Total corrected Relstive Area}{Total responce factor}$ (S4)

**Quality assurance and quality control.** To ensure the reliable quantification of the target compounds, strict quality control steps were carried out. All the laboratory glassware used were fully cleaned and heated for 5 h at 450 °C. Furthermore, prior to use, the glassware would be rinsed with acetone thrice and n-hexane thrice, respectively. A procedural blank (the entire clean-up including ASE but without sample) and a solvent blank (solvents used evaporated to the final volume of 1 mL) were prepared for each batch of six samples. The results indicated that low levels (<0.5 ng μL^-1^) of C_10_- and C_11_-CPs could be identified in some blanks. The method detection limit was defined as three times the standard deviation of laboratory blanks and was 7.8 ng/g. To ensure the validity of the standard curves, SCCP standards containing 55.5% and 63% of chlorine were injected every 20 injections. The results suggested that recoveries of SCCP standards in the spiked laboratory blanks were in the range of 81.1%-86.9%. The relative standard deviation values of the replicates were less than 15%. The recoveries of surrogate standards (^13^C_10_-trans-chlordane) in all fish samples ranged from 69.3% to 95.5%.

**2. Model evaluation and uncertainty**

**Model performance evaluation and validation.** Model validation was necessary to establish confidence in the present model resulting from these efforts. Therefore, we have carried out extensive model performance evaluations and validations via statistical error analysis for modelled and sampled SCCPs concentration data in air, seawater, sediment and seafood (Datasets S1). Model validation metrics include the mean bias (MB), the mean error (ME), the normalized mean bias (NMB) and the normalized mean error (NME). Detailed description of the calculation of each of these metrics have been described previously^4,5^. Table S5 shows that simulated SCCPs concentrations agree well with the observations, with a correlation coefficient of *r* =0.41 (*p*<0.001) for air, *r*=0.23 (*p*<0.001) for soil, and *r* =0.67 (*p*<0.001) for sediment, respectively. Relative poor correlation between simulated and sampled water concentrations is likely caused by small sample size. In addition, 94.44%, 74.68%, 66.67%, and 90.0% of simulated SCCPs concentrations were within a factor of 10 of the corresponding measured value (FAC10) for air, soil, water and sediment, respectively (Fig. S15 and Table S5), thereby demonstrating the reliability of the model. The MB and NMB between simulated and measured SCCPs concentration for all sample size is -3.77 ng·m^−3^ and -11.94% for air, and -20.28 ng·g^−1^ and -44.71% for sediment, respectively, suggesting that the model slightly underestimated SCCPs air and soil concentration at most sampling sites. The model appeared to underestimate SCCP concentrations in soil at most sampling sites with a negative MB (-121.54 ng·g^−1^) and NMB (-52.1%), respectively. However, the comparison results for water concentrations show significant underestimation, the MB and NMB values are -438.74 ng·L^−1^ and -85.02%. Considering that the water samples were collected from intertidal zones, the complex sources^6-8^, the uncertainties of SCCPs in the environmental cycling and pathways in the intertidal zones may lead to the large discrepancy between simulations and observations^9,10^.

**Uncertainties analysis.** Uncertainty analysis shows how a model’s outputs are affected by variation in inputs to reflect uncertainty around the output. We have previously identified the SCCPs emission inventory, physicochemical properties of SCCPs, meteorological conditions, and other factors used in simulating marine food web model as the major contributors to uncertainty in model outputs^1^. Because of complexity and computing time of the CanMETOP model, the Monte Carlo analysis method is no longer suitable for analysing the uncertainty of the simulated concentration. Instead, we used a first-order error propagation approach to calculate the uncertainties in modeled SCCPs concentrations and EDI. The first-order error propagation method was detailed in our previous study^1,5^. Briefly, the estimated uncertainty results were presented by the confidence factors (*Cf*) that span a 95% confidence interval. To obtain the *Cf* for SCCPs concentrations and EDI, one needs to determine the uncertainty information for input parameters, such as coefficient of variation (*CV*) and standard deviation (*SD*). The confidence factor of SCCPs emission inventories was 1.56−1.58 in this study^1^, and the confidence factors for physicochemical properties of SCCPs are listed in Table S6. Moreover, the *CV* for the weight and lipid content of fish were derived from this study, and for lipid content in the diet of fishes the *CV* were derived from literature and listed in Table S7-9. Finally, the uncertainty of SCCP concentrations for air, soil, water, sediment, and fish was estimated with factors of 2.0−2.7, 2.1−2.8, 2.0−6.2, 2.2−3.0, and 2.2−7.4 across the model domain and the uncertainty of EDI for fish consumers with factors of 9.0−11.6.

**3. Food web model**

**SCCPs in benthic invertebrates.** SCCPs concentrations in benthic aquatic invertebrates are established up by the uptake and accumulation through the equilibrium partitioning of SCCPs between the lipids of organism, the organic carbon fraction (OC) of sediments, and the interstitial water^11^, defined by:

$C_{B}\times{d_{L}}/{L_{B}=C_{S}\times d_{OC}/OC}$ (S5)

where *C*_B_ is the SCCPs concentration in benthic invertebrate (ng·g^-1^), *C*_S_ is the SCCPs concentration in sediments (ng·g^-1^), *d*_L_ is the density of lipids of benthos (kg·L^-1^)^12^, *L*_B_ is the lipid fraction of benthos (kg lipid/kg organism)^11,12^, *d*_OC_ is the density of organic carbon fraction of the sediments (kg·L^-1^)^11,12^, and OC is the organic carbon fraction of the sediments (kg organic carbon/kg sediment)^13^. The present study evaluated the contamination of SCCPs in three invertebrate species, including prawn, clam, and oyster, inhabiting the east coast sea of China. The input parameters for modeling benthic invertebrates were presented in Table S7.

**SCCPs in fish.** For fish, chemical uptake comes from water intake and food consumption, and elimination occurs through metabolism, egestion, discharge through gills, and growth dilution^14,15^. The food web model of fishes can be defined as:

${{dC}_{F}}/{dt}=k_{1}C_{W}+k_{D} (P_{i}C_{D,i})-(k_{2}+k_{E}+k_{M}+k_{G})C_{F}$ (S6)

where *C_F_* is the concentration of SCCPs in fish (ng kg^-1^); *k*_1_ is fish intake rate constant of SCCPs from water via gills (L·kg^-1^·day^-1^); *k*_D_ is the rate constant for SCCPs uptake from food intake (kg·(food)^-1^·kg·(fish)^-1^·day^-1^); the constant *P*_i_ is the fraction of the diet constitution of prey item *i*; *k*_2_, *k*_E_, *k*_M_ and *k*_G_ are fish gills removal, egestion, metabolism, growth and dilution SCCPs rate constant (day^-1^), respectively. The calculation of *k*_1_, *k*_2_, *k*_E_, *k*_M_ and *k*_G_ are based on the method of Arnot^16,17^ and Gobas^11^, the specific process is listed below.

In fish, the gill uptake rate constant, *k*_1_, is a function of the gill ventilation rate (*G*_V_, L d^-1^) and the diffusion rate of the chemical across the respiratory surface area,

$k_{1}=(E_{W}\times G_{V})/W$ (S7)

where *E*_W_ is the gill chemical uptake efficiency, and *W* is the wet weight of the organism (kg). *E*_W_ is a function of the octanol-water partition coefficient, *K*_OW_, and in fish can be approximated as,

$E_{W}={(1.85+155/K_{\mathrm{ow}})}^{-1}$ (S8)

*G*_V_ in Eq. (1) is approximately derived from an allometric relationship based on wet weight and dissolved oxygen concentration *D*_ox_ (mg·O_2_/L)^16^,

$G_{V}=(980\times W^{0.65})/D_{\mathrm{ox}}$ (S9)

The elimination rate constant of chemical via the respiratory for fish is expressed as the gill elimination rate constant (day^-1^),

$k_{2}=k_{1}/(P_{B}\times K_{\mathrm{ow}})$ (S10)

where *P*_B_ is the protein content in the fish body.

Previous studies indicate that the fecal elimination rate constant *k*_E_ is approximately 3 to 5 times smaller than the ingestion rate *k*_D_ because the fish food digestion would affect its egestion rate (day^-1^) which is smaller than the feeding rate (day^-1^) in order to maintain^11^. Both *k*_E_ and *k*_D_ are related to the feeding rate. Here, following the studies we choose, *k*_E_ to be a factor of 4 lower than *k*_D_,

$k_{E}=0.25\times k_{D}$ (S11)

The rate constant (day^-1^) for uptake of chemical from food is defined as:

$k_{D}=(F_{D}\times E_{D})/W$ (S12)

Although varying considerably from 0 to 100% in amphipods, mollusks, snails, clams and bivalves and between 0 to 90% in fish, *E*_D_ is approximately 50% for chemicals with a log *K*_ow_ ranging from 6 to 7. Based on the lipid-water two-phase resistance model which hypothesizes that dietary transfer composes transport in aqueous and in lipid (or membrane) phases, we have.

$E_{D}={(5.1\times{10}^{-8}K_{ow}+2)}^{-1}$ (S13)

Fish feeding rates have been described by models of various levels of complexity^18,19^. We use the simple laboratory derived equation based on bioenergetics developed by^20^:

$F_{D}=0.22\times W^{0.85}\times exp(0.06T)$ (S14)

The rate constant due to growth (kg) can be expressed as follows:

$k_{G}=0.0005\times W^{-0.2}$ for temperatures around 10°C (S15)

$k_{G}=0.00251\times W^{-0.2}$ for temperatures around 25°C (S16)

As the contaminants in question are persistent, we assign km a negligible value and expect that the metabolic transformation rate constant *k*_M_ is insignificant relative to other contaminant loss rate constants^11^.

**4. SCCPs level in farmed fish species**

Aquaculture products currently constitute a large portion of the population's diet, with feed comprising the major diet of farmed fish^21^. In contrast to bioaccumulation and biomagnification through the food chain in wild fish, SCCPs present in farmed fish (large yellow croaker) may originate from contaminated feed materials^21-24^. Therefore, the accumulation efficiency for feed-to-fillet transfer can be used to estimate SCCPs level in fish fillets. A simple one-compartment fish biomagnification model derived from Sijm et al.^25^ was employed in this study to predict SCCPs concentrations in feed-fed fish. The model describes the feed-to-fish transfer is defined as:

$C_{fish}\left( t \right)=\frac{\alpha Ft}{k+\gamma}C_{feed}\left( 1-e^{-\left( k+\gamma\right)t} \right)+C_{fish0}e^{-(k+\gamma)t}$ (S17)

where *C*_fish_ is the chemical level in farmed fish (ng g^-1^ wet weight); *C*_feed_ is the chemical level in feed (ng g^-1^ wet weight); *F* is feeding rate (g feed g^-1^ fish d^-1^); *α* is the uptake rate of chemical (%); *C*_fish0_ is initial chemical concentration in fish (ng g^-1^ wet weight); k is the elimination constant (k^-1^); and *γ* is the growth rate (d^-1^).

In the kinetic model simulating the feed-to-fillet transfer of SCCPs in feeds to farmed fish, the SCCPs level in farmed fish through feed intake at a steady state is given by:

$C_{fish}=\delta FtC_{feed}$ (S18)

where 𝛿 is the accumulation efficiency (%), or the net effect of dietary absorption and elimination for salmon. If the feed composition and SCCPs concentrations are known, eq. S17 can be rewritten as:

$C_{fish}=\sum_{i=1}^{n} \delta F_{total}f_{i}C_{feed,i}$ (S19)

where *F*_total_ is a total feed intake rate for farmed salmon (kg feed kg^-1^ fish); *f*_i_ is feed composition (%); and *C*_feed,i_ is the SCCPs concentration level in feed ingredient *i* (ng g^-1^ wet weigh).

Since the health risk of a chemical occurs by ingesting edible part of salmon contaminated by the chemical, the accumulation efficiency for feed-to-fillet transfer is used to estimate SCCPs level in fish fillets. In eq. S19, δ for the feed-to-fillet transfer is 0.47±0.014 ^21^, *F*_total_ is the feed conversion ratio (FCR), which is the amount of feed (in kilograms) required to produce 1 kg of farmed animal (Table S10).

**5. Calculating interprovincial seafood trade flow**

In the standard Leontief and Strout gravity model^26^, the interprovincial trade flows are specified as a function of the total regional outflows, total regional inflows, and transfer cost, which is usually proxied by a spatial friction. Detailed technical specification of the gravity model method is described in previous studies^1,27,28^. The gravity model can be expressed as follows:

$X_{i}^{gh}= \frac{X_{i}^{g} \times X_{i}^{h}}{\sum_{g} X_{i}^{g}} \times Q_{i}^{gh}$ (S20)

where $X_{i}^{gh}$ i represents trade flows of fish species *i* from province *g* to province *h*; $X_{i}^{g}$ is the total outflows of fish species *i* from province g; $X_{i}^{h}$ is the total inflows of fish species *i* to province *h*, and both $X_{i}^{g}$ and$X_{i}^{h}$ are known from statistics (purchased from the Beijing Zhongshang Hua-yan Information Technology Research Institute, <http://www.hyzsyjy.com/>, Fig. S16); $\sum_{g} X_{i}^{g}=\sum_{h} X_{i}^{h}$ holds; $Q_{i}^{gh}$ is the spatial friction of trade flows between province *g* and *h*.

$Q^{gh}$ can be derived from distribution coefficient of transport^27,29^,

$Q^{gh}= \frac{H^{gh}}{\frac{H^{g}H^{h}}{H^{oo}}}$ (S21)

where $H^{gh}$ is the amount of interprovincial commodity *i* flow from province *g* to province *h*; $H^{g}$ is the total amount of commodity *i* outflow from province *g*; $H^{h}$is the total amount of commodity inflow to province *h*; $H^{oo}$ is the total amount of commodity transportation of all provinces. The transport data of goods for every province was obtained from the China Transport Statistical Yearbook (<http://cnki.nbsti.net/CSYDMirror/trade/Yearbook/Single/N2022060165?z=Z014>).

The rationale for selecting the gravity model as the preferred method for approximating interprovincial commodity trade flows, as opposed to other available options, is expounded upon below. First, the gravity model is the most appropriate approach on the basis of available Chinese data. The approaches to construct interregional flow of goods can be identified as survey and non-survey approaches^30^. The survey-based approach identifies interregional trade flows from a collection of primary data by surveys of industries and consumers, while non-survey techniques estimate interregional trade flows by various modification techniques and available data^1,31,32^. The gravity model has become the mainstream non-survey tool to estimate the interregional trade flows, not only for its simplicity, but also because of the fewer data requirements. The feasibility and reliability of this approach have been proven in many studies^33-35^.

Due to the lack of statistical or survey data on inter-provincial seafood transport, the outcomes of this research model were compared to those obtained in existing research^36^ to evaluate the effectiveness of inter-regional transport simulation in this study. We matched the interprovincial transfer of total seafood between this and previous research methods and then conducted regression analysis of the transfer amount (Fig. S17). The results revealed *R*^2^=0.6853, indicating that the simulation value in this study attained a strong correlation with that in the previous study, which verifies the model simulation robustness. Moreover, the trade flow direction in space also revealed that seafood trade basically followed the spatial proximity principle. In order to quantitatively evaluate the model further, trade data collection is required. Subsequently, the accuracy of model can be verified by collected actual trade data in a given year. Consequently, going forward, in order to verify model results, heightened emphasis should be placed on the collection of seafood trade data.

**6. Validation of seafood intake rates**

We compared the major seafood intake data in this study with the consumption data of aquatic products from the China Statistical Yearbook (NBSC, <http://www.stats.gov.cn/>) and the Food and Agriculture Organization (FAO, <https://www.fao.org/faostat/en/#data>). The average daily intake of seafood for 2021 in China was 20.4 g/capita/day in this study, which was approximately 49.8% of the per capita consumption of aquatic products of the Yearbook (38.9 g/capita/day in 2021). This ratio is comparable to that reported by Deng et al.^37^ In addition, average marine fish intake rate was 3.1 g/capita/day in 2021 in this study, slightly lower than FAO (4.5 g/capita/day in 2020). There were no species-specific fish consumption data both in the Yearbook and FAO. Therefore, we further compared the specific fish intake data in this study with the consumption data from the published literatures^1,38-40^. Results show that the seafood intake rates for species predicted in the present study were consistent basically with those reported by literatures (Fig. S18).

**7. Optimization of interprovincial seafood trade flows**

The optimization procedure for trade flows is developed by utilizing the Matlab optimization toolbox, which is based on a multi-objective linear programming^41-44^. The linear programming model takes into account the seafood supply and specific demands of individual provinces, while simultaneously optimizing the transportation cost of the trade flow and minimizing human exposure to SCCP (that is, the objective functions)^45-47^. In the linear programming model, the quantities of consumption for each seafood item were the independent variable, and the transportation cost and exposure level were the coefficients for the independent variable. The constraints for the linear optimization include production and demand of seafood for each province (Fig. S16).

The linear programming model described above is summarized mathematically as follows:

Minimize:

$f_{cost}=\sum_{i=1,j=1,h=1}^{i=11,j=31,h=8} t_{i,j}x_{i,j,h}$ (S22)

$f_{EDI}=\sum_{i=1,j=1,h=1}^{i=11,j=31,h=1} e_{i,j,h}x_{i,j,h}$ (S23)

Subject to:

$x_{i,j}\geq0$ (S24)

$\sum_{i=1,h=1}^{i=11,h=8} x_{i,j,h}$=$X_{i,h}$ (S25)

$\sum_{j=1,h=1}^{j=31,h=8} x_{i,j,h}$=$X_{j,h}$ (S26)

where *f*_cost_ (in RMB) refers to the total transportation cost of the inter-provincial trade in seafoods; *f*_EDI_ (ng kg^-1^ day^-1^) the total seafood consumption EDI in China; *h* is the number of seafood items included in the linear programming analysis; *t*_i,j,h_ is the cost per unit of seafood item *h* between province *i* and province *j*, which were estimated by applying the “cost matrix analysis tool” in ArcGIS while taking the transport cost as the impedance and assuming unit costs of rail transport (RMB tonnes^-1^)^47,48^; *x*_i,j,h_ (tonnes) is the mass of seafood item *h* transfer between provinces; *e*_i,j,h_ is the amount of SCCPs EDI per unit of seafood item *h* from province *i* to province *j*; *X*_i,h_ (tonnes) is the production of seafood item *h* in province *i*; *X*_j,h_ (tonnes) is the total demand of seafood item *h* in province *j*. Eq. S22 represents the minimization of transportation cost. Eq. S23 shows that seafood is preferentially obtained from provinces with low SCCPs pollution.

For calculating the multi-objective optimization problem, different weights were first assigned according to each target's importance^41^. We then weight these functions and take them as a new objective function and present them as a general form:

Minimize:

${F=\lambda_{1}f}_{cost}+\lambda_{2}f_{EDI}$ (S27)

where λ_1_ and λ_2_ is the weight of indicator *f*_cost_ and *f*_EDI_, respectively. The calculation of indicator weight is achieved using Matlab programming^47^.

**8. Concentrations and homologue profiles of SCCPs in seafood samples**

SCCPs were detected in all samples, and detailed information for each species is listed in Table S3. The SCCPs concentrations were shown on a wet weight (ww) basis, lipid weight (lw) basis, and dry weight (dw) basis. The SCCPs concentrations in all seafood ranged from 3.7 to 212.1 ng g^-1^ ww, 363.2 to 17300 ng g^-1^ lw, and 15.8 to 1580.8 ng g^-1^ dw, with average values of 31.7 ± 23.6 ng g^-1^ ww, 2877 ± 2384.1 ng g^-1^ lw, and 164.5 ± 1179.1 ng g^-1^ dw, respectively. The highest concentrations of SCCPs were detected in the clam and oyster samples. Moreover, the relative abundance of SCCPs with different carbon atoms (C_10_–C_13_) were similar in the marine species sampled from the China coastal seas (Fig. S19). An increase trend was observed for SCCPs congener group profiles with the increasing carbon atoms (C_10_ > C_11_ > C_12_ > C_13_) when the average percentages were calculated for all species. Regarding the chlorine content, Cl_7_ and Cl_8_ were the dominant congeners with the average percentages ranging from 6.8%–41.0% (average, 25.21%) and from 6.5%–41.7% (average, 24.39%) of the total SCCPs, respectively.

**9. Concentrations and homologue profiles of SCCPs in fish feed sample**

Table S11 shows that the SCCPs concentrations in five feed samples ranged from 71.9 ng g^-1^ dw to 651.5 ng g^-1^ dw, with the mean of 421.3 ng g^-1^ dw. The concentrations of SCCPs varied among the types of feed materials. SCCPs concentrations also varied among samples of the same type of feed material. Overall, the highest concentration of SCCPs was observed in the traditional feed sample, follow by the ingredient feed. Moreover, the most abundant congener groups of the SCCPs in the feed materials were C_10_ and C_11_, which accounted for approximately 26.7±4.5% and 33.5±6.0% of the total SCCPs, respectively. The predominant chlorine congener groups of SCCPs in the feed materials were Cl_6_ and Cl_7_, which contributed 27.6±6.9% and 16.5±3.1% of the total SCCPs, respectively. The carbon and chlorine congener group patterns of SCCPs in the animal feed materials were consistent with the measured in present study for seafood (Supplementary Section 8).

**Fig. S1.** Production for selected seafood at a 0.5° longitude by 0.5° latitude resolution. (A) hairtail, (B) large yellow croaker, (C) small yellow croaker, (D) sea bass, (E) pomfret, (F) prawn, (G) clam, and (H) oyster.

**Fig. S2.** Modeled SCCPs concentrations in seafood (ng·g^-1^ ww) for different categories. (A) hairtail, (B) large yellow croaker, (C) small yellow croaker, (D) sea bass, (E) pomfret, (F) prawn, (G) clam, and (H) oyster.

**Fig. S3.** Comparison between modelled and measured concentrations of SCCPs for various seafood categories.

**Fig. S4.** Annually averaged SCCPs concentrations from 2008 to 2021. (A) air (ng·m^-3^), (B) soil (ng·g^-1^), (C) water (ng·L^-1^), and D. sediment (ng·g^-1^ ww).

**Fig. S5.** Per capita seafood consumption across China in 2021 (g/day).

**Fig. S6.** Interregional EDI transfers embodied in the seafood trade illustrated as Circos type graph. Numbers in brackets indicate EDIs of SCCPs in each province. The width of each band represents the magnitude of EDI and the band color represents the net inflow of EDI. (A) total seafood, (B) hairtail, (C) large yellow croaker, (D) small yellow croaker, (E) sea bass, (F) pomfret, (G) prawn, (H) clam, and (I) oyster.

**Fig. S7.** SCCPs EDI transfer embodied in interprovincial seafood trade in 2021 in China. Red indicates provinces of net EDI via fish export; blue indicates net EDI via fish import through interprovincial trade. The arrows indicate EDI transfers as defined in the panels.

**Fig. S8.** Modeled SCCPs EDI subject to ‘‘no trade’’ simulation.

**Fig. S9.** The differences of SCCPs EDI between ‘‘trade’’ and ‘‘no trade’’ simulations.

**Fig. S10.** Inter-provincial trade patterns in 2021 for (A) total seafood, (B) hairtail, (C) large yellow croaker, (D) small yellow croaker, (E) sea bass, (F) pomfret, (G) prawn, (H) clam, and (I) oyster.

**Fig. S11.** Optimized inter-provincial trade patterns in 2021 for (A) total seafood, (B) hairtail, (C) large yellow croaker, (D) small yellow croaker, (E) sea bass, (F) pomfret, (G) prawn, (H) clam, and (I) oyster.

**Fig. S12.** Relative differences in provincial SCCPs EDI between optimized trade scenario considering health risk (EDI_optimized_) and current trade scenario non- considering health risk (EDI_current_) for each seafood species, calculated by EDI_RD_ = (EDI_optimized_−EDI_current_)×100/EDI_current_.

**Fig. S13.** Map of the sampling area. A, The data for the base map was derived from the Resource and Environment Science and Data Center of Chinese Academy of Sciences (<http://www.resdc.cn/>). B, Fishing ground distribution data were collected from the National Agricultural Science Data Center (<https://www.agridata.cn/>).

**Fig. S14.** The standard curves based on linearity relationship between calculated chlorination and response factor and used for quantification of SCCPs. Seven-point SCCPs standard curve using SCCPs mixtures with chlorine content between 51.5% and 63%.

**Fig. S15.** Comparison between modeled and measured concentrations of SCCPs in (A) air (ng m^-3^), (B) soil (ng g^-1^ ww), (C) water (ng L^-1^), and (D) sediment (ng g^-1^ ww), respectively.

**Fig. S16.** The spatial distribution of seafood production and consumption in different provinces for 2021 in China.

**Fig. S17.** (A) Inter-provincial trade patterns for total seafood calculated according to Liu et al.^36^, and (B) comparison between inter-provincial seafood trade flow in this study and that obtained according to Liu et al.^36^

**Fig. S18.** Validation of seafood intake rates. (A) total seafood, (B) small yellow croaker, (C) hairtail, and (D) prawn.

**Fig. S19.** Congener group profiles of SCCPs in eight seafood categories collected from ten sampling sites.

**Table S1.** Diet composition of select 5 marine fish species.

| Species | Diet composition | Source |
| --- | --- | --- |
| Hairtail | 90.57% Fish (26.19% Decapterus maruadsi, 10.21% Sardinella jussieu, 9.94% Bregmaceros rarisquamosus, 7.20%Stolephorus heteroloba, 4.34% Trichiurus lepturus, 3.78% Bregmaceros nectabanus, 3.67% Leiognathus bindus, 2.09% Sardinella aurita, 1.80% Leiognathus lineolatus, 1.43% Nemipterus virgatus, 1.31% Trachurus japonicus, 1.28% Stolephorus commersoni, 1.13% Euthynnus alletteratus, 1.11% Alepes kleinii, 15.09% other fish), 2.35% Crustacea (1.21% Acetes chinensis, 1.14% other decapoda), 7.04% Cephalopoda (6.07% Loligo chinensis, 0.97% other cephalopoda) | 49 |
|  | 57.32% Fish (10.33% Stolephorus jussieu, 4.78% Trichiurus lepturus, 4.49% Sardinella jussieu, 4.46% Trachurus japonicus, 2.81% Thryssa mystax, 3.1% Pneumatophorus japonicus, 2.65% Saurida, 1.28% Argyrosomus argentatus, 1.17% Thryssa sp., 0.44% Decapterus maruadsi, 0.44% Larimichthys polyactis, 21.07% other fish), 31.43% Crustacea (11.08% Acanthephyra sp., 5.31% Sergia, 1.83% Solenocera crassicornis, 13.21% other crustacea), 11.1% Cephalopoda (10.99% Loligo chinensis, 0.11% other ) | 50 |
|  | 69.48% Fish (23.26% Bregmaceros rarisquamosus, 10.91% Diaphus sp., 6.13% Bregmaceros nectabanus, 4.01% Trachurus japonicus, 3.39% Larimichthys polyactis, 2.8% Stolephorus, 0.71% Saurida, 0.71% Decapterus maruadsi, 0.69% Argyrosomus argentatus, 0.35% Thryssa dussumieri, 16.52% other fish), 26.02% Crustacea (11.72% Penaeus sp., 4.95% Sergia, 9.35% other), 3.54% Cephalopoda (3.09% Loligo chinensis, 0.45% Sepiida sp.) | 50 |
|  | 79.5% Fish (9.47% Trachurus japonicus, 4.79% Thryssa hamiltonii, 4.52% Trichiurus lepturus, 3.29% Saurida, 1.37% Thryssa dussumieri, 1.13% Sardinella, 0.65% Bregmaceros rarisquamosus, 0.68% Decapterus maruadsi, 0.35% Sardinella, 53.25% other fish), 18.22% Crustacea (1.06% Penaeus, 0.68% Heterocarpoides laevicarina, 16.48% other), 2.03% Cephalopoda (2.03% Loligo chinensis) | 50 |
|  | 93.987% Fish (35.223% Trichiurus japonicus, 8.443% Psenopsis anomala, 5.056% Anguilliformes, 10.16% Auxis sp, 2.247% Apogon lineatus, 5.784% Trachurus japonicus, 1.206% Perciformes, 2.501% Larimichthys polyactis, 3.143% Larimichthys polyactis, 1.3% Decapterus maruaelsi, 1.786% Harpadon nehereus, 1.258% Upeneus bensasi, 1.973% Anago anago), 2.874% Crustaceans (0.931% Euphausia sp, 0.504% Mysidacea sp, 1.89% other), 3.065% Cephalopods (1.59% Loligo edulis, 0.531% Loligo sp, 0.944% other) | 51 |
| Large yellow croaker | 0.56% Euphausiacea (0.2% Mysidacea, 0.36% other), 0.12% Isopoda, 0.12% Amphipoda (0.1% Caprella sp, 0.2% other), 13.13% Decapoda (2.73% Parapenaeopsis hardwickii, 1.43% Latreutes planirostris, 1.06% Solenocera crassicornis, 5.22% other decapoda), 2.74% Stomatopoda (0.55% Oratosquilla inornate, other), 14.68% Crabs (1.17% Portunus trituberculatus, 13.51% Other crabs), 68.61% Fish (9.24% Liza carinatus, 0.52% Bregmaceros macclellandii, 0.30% Larimichthys crocea, 58.55% other fish) | 52 |
|  | 0.62% Lamellibranchia (Pectinidae sp. larva), 0.2% Copepoda (Daphnia sp), 0.29% Amphipoda (0.18% Caprella sp, 0.11% Gammarus sp), 0.06% Mysidacea, 0.18% Euphausiacea (Euphausia pacifica, Pseudeuphausia sinica), 34.8% Natantia (10.20% Solenocera crassicornis, 2.21% Alpheus japonicus, 3.27% Plesionika izumiae, 3.61% Palaemon graviera, 1.93% Acetes chinensis, other), 8.37% Stomatopoda (Oratosquilla oratoria), 5.12% crabs, 50.53 Fish (2.73% Cynoglossus joyneri, 21.50% Harpadon nehereus, 6.26% Apogonichthys lineatus, 0.90% Cynoglossus purpureomaculatus, 1.70% Thryssa kammalensis, 1.60% Benthosema pterotum, 10.68% Acropoma japonicum, 0.94% Engraulis japonicus, 4.22% other) | 53 |
| Small yellow croaker | 63.53% Fish (23.23% Gobioides, 6.09% Johnius belengerii, 5.00% Larimichthys polyactis, 3.28% Zoarces elongatus, 2.28% Setipinna taty, 1.93% Enedrias fangi, 1.46% Apogonichthys lineatus, 1.44% Engraulis japonicus), 29.23% Decapoda (11.60% Alpheus japonicus, 3.67% Palaemon graviera, 3.59% Crangon affinis, 1.76% Metapenaeopsis dalei, 1.45% Leptochela gracilis, 1.3% Eualus sinensis, other), 0.57% Crabs (0.35% Carcinoplax vestitus, 0.15% Pagurus ochotensis, 0.04% Oregonia gracilis), 2.48% Stomatopoda (Oratosquilla oratoria), 0.14% Amphipoda, 0.29% Polychaeta(Glycera chirori), 0.05% Isopoda, 0.88% Sergestidae (Acetes chinensis), 0.58% Euphausiacea, 2.11% Mysidacea, 0.14% Copepoda (0.11% Calanus sinicus, 0.02% Labidocera euchaeta) | 54 |
|  | 18.8% Fish (5.9% Sebastiscus marmoratus, 2.0% Harpodon nehereus, 4.2% Engraulis japonicus, 3.6% Chaeturichthys hexanema, 3.2% other), 61.5% Shrimps (36.2% Acetes chinensis, 16.1% Leptochela gracilis, 3.3% Palaemon graviera, 1.9% Alpheus distinguendus, 1.2% Solenocera crassicornis, 1.0% Alpheus japonicus, 2.0% other), 3.3% Crabs (2.7% Charybdis japonica, 0.5% Petrolisthes japonicus, 0.2% Unidentified crabs), 4.4% Amphipoda (3.6% Gammaridae, 0.8% Caprellidae), 5.5% Isopoda (Cirolana japonensis), 0.2% Polychaete (Nereis succinea), 1.0% Cephalopoda (Loligo japonica), 0.2% Alga, 5.0% Unidentified species | 55 |
|  | 59.97% Fish (39.05% Anchovy, 0.54% Apogonichthys lineatus, 3.03% Pennahia argentata, Sebastiscus marmoratus, other unidentified fish), 24.76% shrimps (0.59% Acetes chinensis, 3.17% Alpheus distinguendus, 6.61% Leptochela gracilis, 6.23% Alpheus japonicus, 1.78% Latreutes anoplonyx, other), 0.37% crabs (0.36% Charybdis bimaculate, 0.01% unidentified crabs), 4.26% Amphipoda (4.00% Amoelisca sp, 0.23% Gammarus sp., 0.02% Monoclodes sp.), 0.01% Copepoda, (Calanidae sp.), 0.64% Euphausiacea (Euphausia pacifica), 0.31% Stomatopoda (Oratosquilla oratoria), 0.09% Mysidacea, 0.04% Isopoda (Tianais sp.), 0.01% Bivalvia (Dosinia japonica), 0.69% Polychaeta (Nereis succinea), 7.48% Cephalopoda (7.42% Loligo sp., 0.06% Sepiola birostrata), 1.39% other | 56 |
|  | 0.14% Copepoda, 0.58% Euphausiacea (Euphausia pacifica), 0.83% Acetes chinensis, 2.11% Mysidacea, 0.10% Amphipoda, 0.032% Polychaeta, 29.23% Deacpoda (11.60% Alpheus japonicus, 3.67% Palaemon graviera, 3.59% Crangon affinis, 1.45% Leptochela gracilis), 2.48% Stomatopoda (Oratosquilla oratoria), 63.53% Fish (16.6% Chaeturichthys hexanema, 8.96% Chaeturichthys stigmatias, 6.09% Johnius belengeri, 5.00% Small yellow croaker, 1.44% Engraulis japonicus, 25.44% other) | 57 |
| Sea bass | 0.20% Deacpoda (Alpheus japonicus), 0.03% Crab, 99.78% Fish (3.48% Chaeturichthys hexanema, 3.71% Platycephalus indicus, 73.06% Liparis tanakae, 18.46% Larimichthys polyactis) | 57 |
|  | 24.8% Mysids (10.6% Archaeomysis spp., 0.6% Nipponomysis spp., 0.4% Orientomysis japonica, 13.3% Unidentified mysids), 0.4% Amphipods, 0.7% Isopods, 74.1% Polychaetes | 58 |
|  | 23.4% Oratosquilla oratoria, 11.56% Engraulis japonicus, 8.15% Setipinna taty, 9.19% Coilia mystus, 4.37% Pseudosiaena polyactis, 2.85% Coooichtys lucidus, 5.72% C. niveatus, 2.18% Trichiurus muticus, 0.98% Stromatroides argenteus, 1.56% Chaetuichthys stigmatias, 2.33% Acanthogobius hasta, 1.0% Trachypenaeus curvirostris, 2.25% Palaemon gnavieri, 1.76% Alpheus distinguendus, 0.58% Lateolabrax japonicus, other | 59 |
| Pomfret | Amphipods (12.5% Phronima sedentaria, 8.1% Platyscelus ovoides, 0.3% Platyscelus armatus, 2.7% Brachyscelus crusculum, 0.5% Unidentified), 0.3% Euphausiids, Cephalopods (19.2% Onychoteuthis borealijaponica, 7.2% Gonatopsis borealis, 3.1% Gonatus pyros, 2.3% Gonatus, 10.5% Berryteuthis anonychus, 0.4% Unidentified and other), Fish (15.9% Bathylagus sp., 7.1% Maurolicus imperatorius, 0.3% Lampanyctus sp, 1.3% Lampanyctus sp, 0.2% Paralepididae, 2.6% Scombridae, 2.5% Gempylidae, 2.9% Unidentified) | 60 |
|  | 6.6% (0.5% Hyperia medusarum, 0.3% Hyperia glaba, 4.6% Phronima sedentaria, 0.2% Phronima spp., 0.8% Primno abyssalis, 0.2% Unidentified), 0.7% Euphausiids (Euphausia pacifica), 0.1% Decapods, 0.1% Crustaceans, 63.2% Cephalopods, Gonatids (10.9% Gonatus middendorffi, 3.3% Gonatus berryi, 1.5% Gonatus onyx, 0.1% Gonatus madokai, 0.4% Gonatus pyros, 0.7% Gonatus spp., 14.7% Gonatopsis borealis, 14.5% Berryteuthis anonychus), 17% Unidentified and Others | 61 |

**Table S2.** The SCCPs concentrations in different seafood products from the Chinese coastal seas.

| Common name | Scientific name | Family | Mean size (cm) | Dietary habit |
| --- | --- | --- | --- | --- |
| Hairtail | Trichiurus lepturus | Trichiuridae | 69.3-107.6 | Predatory |
| Large Yellow Croaker | Larimichthys crocea | Sciaenidae | 25.7-35.1 | Predatory |
| Small Yellow Croaker | Larimichthys polyactis | Sciaenidae | 14.6-20.8 | Predatory |
| Sea Bass | Lateolabrax japonicus | Moronidae | 36.2-51.3 | Predatory |
| Pomfret | Pampus argenteus | Stromateidae | 15.9-21.7 | Predatory |
| Prawn | Penaeus chinensis | Penaeidae | 9.2-15.2 | Omnivorous |
| Clam | Mactra chinensis | Mactridae | 4.6-7.2 | Filter-feeder |
| Oyster | Crassostrea gigas | Ostreidae | 8.9-15.4 | Filter-feeder |

**Table S3.** The SCCPs concentrations in different seafood products from the Chinese coastal seas.

| Fishing grounds | Species | Sample size (n) | SCCPs (ng g^-1^ ww) | | SCCPs (ng g^-1^ dw) | | SCCPs (ng g^-1^ lw) | |
| --- | --- | --- | --- | --- | --- | --- | --- | --- |
|  |  |  | Mean | SD ^a^ | Mean | SD ^a^ | Mean | SD ^a^ |
| Liaodong Bay fishing ground | Hairtail | 5 | 24.31 | 10.5 | 106.08 | 49.11 | 4652.15 | 1518.8 |
|  | Large yellow croaker | 5 | 8.7 | 2.15 | 33.37 | 7.18 | 480.33 | 153.01 |
|  | Small yellow croaker | 5 | 36.55 | 10.83 | 141.29 | 42.34 | 2917.18 | 498.45 |
|  | Sea bass | 5 | 15.61 | 9.66 | 73.25 | 44.96 | 3636.63 | 1681.13 |
|  | Pomfret | 5 | 26.62 | 6.07 | 103.77 | 22.76 | 2928.13 | 628.67 |
|  | Prawn ^b^ | - | 63.95 |  | 240.55 |  | 5906.45 |  |
|  | Clam ^b^ | - | 106.84 |  | 699.01 |  | 8004.26 |  |
|  | Oyster ^b^ | - | 51.97 |  | 356.97 |  | 3594.84 |  |
| Luanhekou fishing ground | Hairtail | 5 | 22.42 | 7 | 97.66 | 34.64 | 2384.53 | 803.27 |
|  | Large yellow croaker | 5 | 12.07 | 8.36 | 53.39 | 36.07 | 1204.1 | 548.6 |
|  | Small yellow croaker | 5 | 48.64 | 12.35 | 181.25 | 53.23 | 2234.42 | 828.65 |
|  | Sea bass | 5 | 18.99 | 10.17 | 91.77 | 47.86 | 3246.44 | 2284.15 |
|  | Pomfret | 5 | 22.9 | 5.75 | 116.46 | 28.97 | 2708.41 | 703.85 |
|  | Prawn ^b^ | - | 71.49 |  | 367.1 |  | 3876.49 |  |
|  | Clam ^b^ | - | 51.03 |  | 570.53 |  | 7204.25 |  |
|  | Oyster ^b^ | - | 67.41 |  | 684.75 |  | 6311.52 |  |
| Bohai Bay fishing ground | Hairtail | 5 | 32.57 | 13.86 | 141.41 | 61.54 | 4511.77 | 885.3 |
|  | Large yellow croaker | 5 | 53.68 | 17.48 | 228.05 | 69.61 | 3276.99 | 1069.18 |
|  | Small yellow croaker | 5 | 38.09 | 9.3 | 155.29 | 42.25 | 2491.94 | 485.24 |
|  | Sea bass | 5 | 22.47 | 8.17 | 105.4 | 37.49 | 2435.9 | 1383.35 |
|  | Pomfret | 5 | 29.16 | 10.42 | 153.97 | 53.42 | 2905.87 | 854.96 |
|  | Prawn ^b^ | - | 72.86 |  | 650.94 |  | 12529.42 |  |
|  | Clam ^b^ | - | 131.49 |  | 874.23 |  | 7952.44 |  |
|  | Oyster ^b^ | - | 118.54 |  | 822.03 |  | 8470.04 |  |
| Yanwei fishing ground | Hairtail | 5 | 30.97 | 14.62 | 135.45 | 61.05 | 6235.64 | 1442.65 |
|  | Large yellow croaker | 5 | 46.24 | 7.85 | 227.25 | 75.61 | 2110.77 | 1317.23 |
|  | Small yellow croaker | 5 | 44.04 | 17.35 | 220.86 | 92.11 | 1176.66 | 526.46 |
|  | Sea bass | 5 | 29.63 | 23.68 | 139.36 | 110.8 | 3040.35 | 2569.16 |
|  | Pomfret | 5 | 34.28 | 14.33 | 131.94 | 54.83 | 2687.95 | 562.62 |
|  | Prawn ^b^ | - | 47.6 |  | 567.79 |  | 7986.54 |  |
|  | Clam ^b^ | - | 86.08 |  | 601.41 |  | 10369.06 |  |
|  | Oyster ^b^ | - | 84.81 |  | 636.09 |  | 7921.97 |  |
| Haizhou Bay fishing ground | Hairtail | 5 | 18.86 | 3.89 | 89.27 | 19.71 | 3790.23 | 801.53 |
|  | Large yellow croaker | 5 | 64.94 | 27.45 | 240.71 | 102.25 | 1853.13 | 728.5 |
|  | Small yellow croaker | 5 | 36.87 | 10.97 | 183.34 | 55.05 | 1978.59 | 518.35 |
|  | Sea bass | 5 | 23.24 | 7.29 | 107.92 | 34.22 | 2483.62 | 801.99 |
|  | Pomfret | 5 | 33.81 | 11.09 | 129.29 | 37.99 | 2150.43 | 498.94 |
|  | Prawn ^b^ | - | 39.54 |  | 256.27 |  | 6598.19 |  |
|  | Clam ^b^ | - | 106.11 |  | 1026.57 |  | 10757.2 |  |
|  | Oyster ^b^ | - | 53.4 |  | 414.04 |  | 5001.26 |  |
| Lvsi fishing ground | Hairtail | 5 | 18.3 | 11.95 | 75.64 | 48.97 | 3616.29 | 258.06 |
|  | Large yellow croaker | 5 | 50.32 | 10.72 | 197.11 | 43.39 | 1647.95 | 368.28 |
|  | Small yellow croaker | 5 | 38.6 | 10.45 | 195.22 | 59.7 | 2465.03 | 828.86 |
|  | Sea bass | 5 | 10.79 | 4.6 | 47.14 | 19.37 | 1130.88 | 458.48 |
|  | Pomfret | 5 | 42.22 | 9.39 | 191.97 | 36.41 | 2249.96 | 192.35 |
|  | Prawn ^b^ | - | 56.61 |  | 428.57 |  | 12139.78 |  |
|  | Clam ^b^ | - | 212.12 |  | 1580.86 |  | 17300.04 |  |
|  | Oyster ^b^ | - | 39.32 |  | 234 |  | 5277.83 |  |
| Zhoushan fishing ground | Hairtail | 5 | 11.97 | 5.46 | 51.7 | 18.76 | 2209.14 | 209.48 |
|  | Large yellow croaker | 5 | 45.18 | 14.25 | 177.35 | 60.12 | 1526.56 | 398.39 |
|  | Small yellow croaker | 5 | 28.48 | 12.67 | 127.84 | 54.31 | 1719.5 | 855.95 |
|  | Sea bass | 5 | 6.04 | 2.72 | 25.65 | 11.34 | 582.75 | 290.84 |
|  | Pomfret | 5 | 23.49 | 7.71 | 96.57 | 33.04 | 1534.03 | 351.6 |
|  | Prawn ^b^ | - | 35.37 |  | 211.52 |  | 3764.85 |  |
|  | Clam ^b^ | - | 47.27 |  | 310.7 |  | 3668.77 |  |
|  | Oyster ^b^ | - | 58.97 |  | 837.63 |  | 13636.58 |  |
| Wentai fishing ground | Hairtail | 5 | 21.35 | 12.31 | 110.38 | 57.96 | 3636.07 | 1119.76 |
|  | Large yellow croaker | 5 | 30.53 | 8.73 | 128.39 | 34.82 | 1304.75 | 423.49 |
|  | Small yellow croaker | 5 | 23.88 | 7.95 | 112.3 | 35.78 | 2346.77 | 1211.42 |
|  | Sea bass | 5 | 12.36 | 8.3 | 57.58 | 39.65 | 1361.41 | 800.24 |
|  | Pomfret | 5 | 20.56 | 5.2 | 104.26 | 39.33 | 1252.21 | 141.35 |
|  | Prawn ^b^ | - | 31.69 |  | 268.16 |  | 7497.64 |  |
|  | Clam ^b^ | - | 57.16 |  | 848.89 |  | 13680.77 |  |
|  | Oyster ^b^ | - | 65.62 |  | 709.96 |  | 6288.08 |  |
| Mindong fishing ground | Hairtail | 5 | 14.33 | 5.68 | 61.75 | 20.82 | 2433.12 | 446.73 |
|  | Large yellow croaker | 5 | 39.76 | 12.41 | 158.63 | 45.48 | 2863.3 | 1187.3 |
|  | Small yellow croaker | 5 | 20.57 | 4.42 | 90.29 | 15.38 | 1501.16 | 411.65 |
|  | Sea bass | 5 | 8.63 | 3.49 | 40.9 | 16.27 | 873.16 | 322.79 |
|  | Pomfret | 5 | 14.89 | 3.27 | 61.48 | 12.42 | 1198.74 | 266.52 |
|  | Prawn ^b^ | - | 25.06 |  | 128.95 |  | 1909.33 |  |
|  | Clam ^b^ | - | 46.16 |  | 492.05 |  | 4161.6 |  |
|  | Oyster ^b^ | - | 43.16 |  | 282.35 |  | 3886.27 |  |
| Minzhong fishing ground | Hairtail | 5 | 12.59 | 8.56 | 68.22 | 52.54 | 2484.55 | 760.65 |
|  | Large yellow croaker | 5 | 25.61 | 10.98 | 105.43 | 44.83 | 1244.23 | 737.46 |
|  | Small yellow croaker | 5 | 19.1 | 7.18 | 81.69 | 33.74 | 1335.13 | 479.16 |

Note: a) SD - standard deviation. b) As a result of the limited sample size, the five individual samples were combined and uniformly blended to create a composite sample for subsequent analyses.

**Table S4.** Two most abundant isotopes of SCCPs congeners used for quantification and identification.

| SCCPs congeners | Quantification isotope | Identification isotope |  | SCCPs congeners | Quantification isotope | Identification isotope |
| --- | --- | --- | --- | --- | --- | --- |
| C_10_H_17_Cl_5_ | 279 | 277 |  | C_12_H_21_Cl_5_ | 307 | 305 |
| C_10_H_16_Cl_6_ | 313 | 315 |  | C_12_H_20_Cl_6_ | 341 | 343 |
| C_10_H_15_Cl_7_ | 347 | 349 |  | C_12_H_19_Cl_7_ | 375 | 377 |
| C_10_H_14_Cl_8_ | 381 | 383 |  | C_12_H_18_Cl_8_ | 409 | 411 |
| C_10_H_13_Cl_9_ | 415 | 417 |  | C_12_H_17_Cl_9_ | 445 | 443 |
| C_10_H_12_Cl_10_ | 449 | 451 |  | C_12_H_16_Cl_10_ | 479 | 477 |
| C_11_H_19_Cl_5_ | 293 | 291 |  | C_13_H_23_Cl_5_ | 319 | 321 |
| C_11_H_18_Cl_6_ | 327 | 329 |  | C_13_H_22_Cl_6_ | 355 | 357 |
| C_11_H_17_Cl_7_ | 361 | 363 |  | C_13_H_21_Cl_7_ | 389 | 391 |
| C_11_H_16_Cl_8_ | 395 | 397 |  | C_13_H_20_Cl_8_ | 423 | 425 |
| C_11_H_15_Cl_9_ | 429 | 431 |  | C_13_H_19_Cl_9_ | 459 | 457 |
| C_11_H_14_Cl_10_ | 463 | 465 |  | C_13_H_18_Cl_10_ | 493 | 491 |
| ε-HCH | | |  | ISTD | 255 | 257 |
| ^13^C_10_-trans-chlordane | | |  | Surrogate | 420 | 418 |

**Table S5.** Correlation coefficient, FAC, bias, and error statistics for comparison between modeled and measured SCCPs concentrations for air (ng m^-3^), soil (ng g^-1^), water (ng L^-1^), and sediment (ng g^-1^ ww).

| N | N | r | FAC2 (%) | FAC5 (%) | FAC10 (%) | MB | ME | NMB (%) | NME (%) |
| --- | --- | --- | --- | --- | --- | --- | --- | --- | --- |
| Air | 755 | 0.41 | 50.73 | 84.64 | 94.44 | -3.77 | 20.34 | -11.94 | 64.38 |
| Soil | 237 | 0.23 | 25.32 | 57.81 | 74.68 | -121.54 | 187.42 | -52.1 | 80.33 |
| Water | 21 | 0.37 | - | 47.62 | 66.67 | -438.74 | 438.79 | -85.02 | 85.03 |
| Sediment | 260 | 0.67 | 29.23 | 67.31 | 90 | -20.28 | 31.05 | -44.71 | 68.45 |

**Table S6.** Physicochemical properties of SCCPs.^62-66^

| Parameters | Value | *Cf* | Parameters | Value | *Cf* |
| --- | --- | --- | --- | --- | --- |
| Molecular mass (g mol^−1^) | 377 | 1 | Degradation rate in sediment (s^−1^) | 8.82×10^−9^ | 2 |
| Molar volume (cm^3^ mol^−1^) | 250.3 | 1 | Liquid vapor pressure (Pa) | 2.27×10^−4^ | 1.5 |
| Melting point (^o^C) | 10.0 | 1 | Water solubility (g m^−3^) | 0.0625 | 1.5 |
| Entropy of Fusion (J mol^−1^ K^−1^) | 63.35 | 1 | Log K_ow_ (25 ^o^C)* | 6.4 | 1.5 |
| Degradation rate in air (s^−1^) | 3.6×10^−7^ | 2 | Log K_oa_ (25 ^o^C)* | 9.3 | 1.1 |
| Degradation rate in soil (s^−1^) | 2.198×10^−8^ | 2 | Log H (25 ^o^C)* (Pa m^3^ mol^−1^) | 6.32 | 1.5 |
| Degradation rate in water (s^−1^) | 1.76×10^−8^ | 2 |  |  |  |

* K_ow_ and K_oa_ and H are octanol-water and octanol-air partition coefficient, respectively. H is Henry’s law constant. *Cf* is confidence factor obtained from literature.

**Table S7.** Input parameters for modeling SCCPs concentrations in benthic invertebrates.

| Species | Lipid fraction of benthos (Lb, %) | Organic carbon fraction of sediments (OC) | Reference |
| --- | --- | --- | --- |
| Prawn (*Penaeus* *chinensis*) | 3.8±0.5 | 0.015 | 67,68 |
| Clam *(Mactra chinensis*) | 4.2 | 0.015 | 68,69 |
|  | 7.9 | 0.014±0.0006 | 68-72 |
|  | 4.3 | 0.015 | 67,68,72 |
| Oyster (*Crassostrea gigas*) | 15.7±3.2 | 0.1023±0.169 | 68,71,72 |

**Table S8.** Input parameters used in the marine food web.

| Parameter | Value | Definition | Reference |
| --- | --- | --- | --- |
| C_WT_ (ng L^−1^) | - | SCCP concentration in water | from CanMETOP |
| C_S_ (ng g^−1^) | - | SCCP concentration in sediment | from CanMETOP |
| C_A_ (ng kg^−1^) | - | SCCP concentration in zooplankton | from CanMETOP |
| C_B_ (ng g^−1^) | - | SCCP concentration in benthic invertebrate | from CanMETOP |
| BCF (m^3^ kg^−1^) | 1−7.6 | Bioconcentration factors | 73 |
| T (℃) | 12 | Mean annual water temperature | 72 |
| δ (kg m^−3^) | 1025 | Density of phytoplankton | 72 |
| d_L_ (kg L^−1^) | 0.9 | Density of lipids for benthos | 11 |
| d_OC_ (kg L^−1^) | 0.9 | Density of organic carbon | 11 |
| D_OX_ (mg L^−1^) | 7.1 | Dissolved oxygen concentration | 11 |

**Table S9.** Lipid content of the composition of seafood’s diet.

| No. | Species | Lipid (%) | CV (%) | References | No. | Species | Lipid (%) | CV (%) | References |
| --- | --- | --- | --- | --- | --- | --- | --- | --- | --- |
| 1 | Acanthogobius hasta | 3.32 | 58.5 | 74,75 | 66 | Solenocera crassicornis | 1.11 | 76.1 | 84,140 |
| 2 | Anchovy | 2.9 | 31.5 | 76-81 | 67 | Alpheus japonicus | 3.73 | 16.4 | 138 |
| 3 | Anguilliformes | 8.12 | 57 | 7,82 | 68 | Crangon affinis | 1.7 |  | 84 |
| 4 | Apogon lineatus | 3.8 | 29.8 | 83,84 | 69 | Eualus sinensis | 1.4 |  | 141 |
| 5 | Apogonichthys lineatus | 3.3 | 12.1 | 85 | 70 | Hyperia glaba | 1.04 | 41.3 | 142 |
| 6 | Argyrosomus argentatus | 6.26 | 4 | 86 | 71 | Hyperia medusarum | 1.4 | 27.7 | 142 |
| 7 | Auxis sp. | 4.95 | 4.3 | 87 | 72 | Latreutes anoplonyx | 0.24 | 42.2 | 143 |
| 8 | Bathylagus sp. | 3.17 | 51.9 | 88-90 | 73 | Metapenaeopsis dalei | 0.94 | 5.6 | 97,113 |
| 9 | Benthosema pterotum | 5.63 | 55 | 91 | 74 | Oratosquilla oratoria | 2.73 | 9.1 | 75, 84 |
| 10 | Chaeturichthys stigmatias | 4.75 | 10.4 | 92 | 75 | Palaemon graviera | 2.71 | 61.3 | 84,98,100 |
| 11 | Chaeturichthys hexanema | 4.2 | - | 93 | 76 | Parapenaeopsis hardwickii | 1.24 | 64 | 100,102 |
| 12 | Coilia mystus | 3.65 | 25.4 | 94-97 | 77 | Phronima sedentaria | 3.3 |  | 144 |
| 13 | Collichthys niveatus | 6 | 4.5 | 98 | 78 | Phronima spp. | 5.91 | 30.3 | 145 |
| 14 | Collichthys lucidus | 2.39 | 45.2 | 99-103 | 79 | Primno abyssalis | 1.11 |  | 146 |
| 15 | Cynoglossus joyneri | 0.38 | - | 83 | 80 | Solenocera crassicornis | 1.7 |  | 84 |
| 16 | Cynoglossus purpureomaculatus | 0.56 | 32.1 | 102 | 81 | Trachypenaeus curvirostris | 0.39 | 10.2 | 147 |
| 17 | Decapterus maruadsi | 1.25 | 61.3 | 103,104 | 82 | Decapoda | 2.48 | 85.1 |  |
| 18 | Diaphus sp. | 5 | 35.3 | 91,105 | 83 | Charybdis bimaculate | 4.3 |  | 84 |
| 19 | Enedrias fangi | 4.86 | 14.4 | 75 | 84 | Charybdis japonica | 1.22 | 71.6 | 75,97,100,102 |
| 20 | Engraulis japonicus | 4.42 | 60.6 | 106 | 85 | Oregonia gracilis | 5.9 | - | 84 |
| 21 | Euthynnus alletteratus | 2.79 | 36.5 | 107 | 86 | Pagurus ochotensis | 1.3 | - | 84 |
| 22 | Harpodon nehereus | 1.71 | 53.2 | 95,97,100,  108 | 87 | Portunus trituberculatus | 3.34 | 56.8 | 84,100,114 |
| 23 | Johnius belengeri | 1.06 | 17.9 | 102 | 88 | Crabs | 2.58 | 74.8 |  |
| 24 | Lampanyctus sp. | 9.85 | 29.9 | 109,110 | 90 | Labidocera euchaeta | 1.2 |  | 142 |
| 25 | Larimichthys crocea | 11.18 | 68.9 | 111 | 91 | Calanus sinicus | 0.95 | 81.8 | 142 |
| 26 | Larimichthys polyactis | 2.15 | 33.8 | 95,100 | 92 | Calanidae sp. | 11.3 | 22.1 | 148 |
| 27 | Lateolabrax japonicus | 3.65 | 49.1 | 7,82,92,98,  100,112 | 93 | Copepoda | 1.03 | 55 | 142 |
| 28 | Leiognathus bindus | 1.8 | 3.5 | 113,114 | 94 | Lamellibranchia | 3.17 | 47.9 | 149 |
| 29 | Leiognathus lineolatus | 1.26 | 2.3 | 115 | 95 | Pectinidae | 0.63 | - | 150 |
| 30 | Liparis tanakae | 1.5 | 1.3 | 116 | 96 | Bivalvia | 2.23 | 33.9 | 84,98 |
| 31 | Liza carinatus | 3.7 | 91.9 | 7,102, | 97 | Berryteuthis anonychus | 1 | 10 | 151 |
| 32 | Nemipterus virgatus | 2.44 | 66.7 | 100,102,  103,108 | 98 | Gonatopsis borealis | 2 | 0.2 | 152 |
| 33 | Pennahia argentata | 0.56 | - | 103 | 99 | Gonatus sp. | 5.3 | 28.7 | 153 |
| 34 | Perciformes | 2.35 | 27.9 | 117,118 | 100 | Gonatus berryi | 8.9 |  | 153 |
| 35 | Platycephalus indicus | 2.33 | 48.7 | 83,92 | 101 | Loligo chinensis | 2.14 | 8.6 | 95,100 |
| 36 | Pneumatophorus japonicus | 6.92 | 18.7 | 86,98 | 102 | Loligo edulis | 1.36 | 2.6 | 113 |
| 37 | Psenopsis anomala | 4.12 | 69.3 | 86,103 | 103 | Loligo japonica | 2.9 | - | 84 |
| 38 | Sardinella | 4.19 | 80.3 | 82,119,120 | 104 | Onychoteuthis borealijaponica | 0.33 | 65.4 | 154 |
| 39 | Sardinella aurita | 2.5 | - | 108 | 105 | Sepiola birostrata | 2 | - | 121 |
| 40 | Sardinella jussieu | 1.97 | 28 | 97,108 | 106 | Cephalopoda | 2.8 | 65.6 | 84 |
| 41 | Saurida | 1.51 | 27.2 | 108,113 | 107 | Mollusca | 0.54 |  | 82 |
| 42 | Scombridae | 8.3 | - | 84 | 108 | Squid (Loligo tagoi) | 1.3 | 17 | 108 |
| 43 | Sebastiscus marmoratus | 1.84 | - | 121 | 109 | sepia pharaonis | 5.17 | 6.2 | 98 |
| 44 | Setipinna taty | 11.26 | - | 99 | 110 | octopus vulgaris | 5.33 | 4.9 | 98 |
| 45 | Small yellow croaker | 3.08 | - | 95,100 | 111 | Octopus | 1.52 |  | 95,100 |
| 46 | Stolephorus. sp | 2.38 | 47.2 | 114,115,122 | 112 | Loligo | 1.45 | 73.1 | 121 |
| 47 | Stolephorus commersoni | 3.05 | 23.6 | 119,123 | 113 | Amoelisca sp | 0.08 | 3.9 | 155 |
| 48 | Thryssa dussumieri | 7.2 |  | 95,100 | 114 | Caprella sp | 7.35 | 43.3 | 156 |
| 49 | Thryssa hamiltonii | 3.33 |  | 124 | 115 | Gammarus sp | 2.72 |  | 142 |
| 50 | Thryssa kammalensis | 5.59 | 44.5 | 100,102 | 116 | Monoclodes sp. | 2.89 |  | 157 |
| 51 | Thryssa sp. | 4.84 | 40.4 | 122,124 | 117 | Phronima sedentaria | 0.8 | 62.5 | 158 |
| 52 | Trichiurus japonicus | 7.33 | 8 | 86 | 118 | Platyscelus armatus | 5.2 | 39.7 | 142 |
| 53 | Trichiurus lepturus | 4.72 | 36.2 | 7,84,100 | 119 | Amphipoda | 3.69 | 84.9 | 142,155,156,  157,158 |
| 54 | Trichiurus muticus | 2.85 | 22.3 | 121 | 120 | Cirolana japonensis | 3.01 | 9.3 | 159 |
| 55 | Upeneus bensasi | 0.95 | 8.9 | 113 | 121 | Isopoda | 3.01 | 9.3 | 159 |
| 56 | Pisces | 3.65 | 0.68 |  | 121 | Oratosquilla inornate | 1.5 | 9.4 | 108 |
| 57 | Acanthephyra sp. | 2.28 | 45.9 | 90,125 | 122 | Oratosquilla oratoria | 1.61 | 26.8 | 86,108 |
| 58 | Acetes chinensis | 2.08 | 28.8 | 126,127 | 123 | Stomatopoda | 1.564 | 31.8 | 86,108 |
| 59 | Decapoda | 1.28 | 44 | 121 | 124 | Glycera chirori | 1.9 |  | 142 |
| 60 | Euphausia sp. | 2.01 | 63.6 | 121,128,129 | 125 | Nereis succinea | 1.11 | 44.1 | 90 |
| 61 | Euphausia pacifica | 2.47 | 42.7 | 130-132 | 126 | Nereis succinea | 1.19 | 39.5 | 90 |
| 62 | Mysidacea sp. | 13.15 | 71.6 | 133-137 | 127 | Squilla orarotia | 5 | 13.6 | 98 |
| 63 | Penaeus | 2.25 | 52.5 | 75,86,113,138 | 128 | Crassostrea gigas | 3.67 | 7.6 | 98 |
| 64 | Pseudeuphausia sinica | 0.52 | 10.9 | 139 | 129 | Polychaete | 3.29 | 58.8 | 90,98,142 |
| 65 | Sergia | 1.57 | 30.9 | 125 |  |  |  |  |  |

**Table S10.** Feed conversion ratio for the farmed large yellow croaker.

|  | Range | Mean | CV (%) | Reference |
| --- | --- | --- | --- | --- |
| Traditional feed | 5.0-8.5 | 7 | 19.5 | 160-162 |
| Ingredient feed | 1.4-3 | 1.8 | 24.6 | 160-162 |

**Table S11.** Concentrations (ng g^-1^ ww) of SCCPs in feed material samples.

| Sample | Lipid content (%) | SCCPs |
| --- | --- | --- |
| Traditional feed | 8.2 | 71.9 |
| Traditional feed | 30.2 | 135.4 |
| Ingredient feed | 6.6 | 651.5 |
| Ingredient feed | 7.5 | 649.2 |
| Ingredient feed | 7.4 | 598.6 |

**Dataset S1 (Excel).** Comparisons between modeled and measured SCCPs concentrations in air, soil, water, and sediment.

**SI References**

1. Song, S. et al. Assessing Safety of Market-Sold Fresh Fish: Tracking Fish Origins and Toxic Chemical Origins. *Environ. Sci. Technol.* 56, 11256–11265 (2022).
2. Reth, M., Zencak, Z. & Oehme, M. New quantification procedure for the analysis of chlorinated paraffins using electron capture negative ionization mass spectrometry. *J. Chromatogr. A* 1081, 225-231 (2005).
3. Reth, M. et al. Short- and medium-chain chlorinated paraffins in biota from the European Arctic-differences in homologue group patterns. *Sci. Total Environ.* 367, 252–260 (2006).
4. Chang, J. & Hanna, S. Air quality model performance evaluation. *Meteorol. Atmos. Phys.* 87, 167–196 (2004).
5. Huang, T. et al. Human exposure to polychlorinated biphenyls embodied in global fish trade. *Nat. Food* 1, 292–300 (2020).
6. Zhao, N. et al. Short-chain chlorinated paraffins in soil, sediment, and seawater in the intertidal zone of Shandong Peninsula, China: Distribution and composition. *Chemosphere* 220, 452–458 (2019).
7. Huang, Y. et al. Bioaccumulation and biomagnification of short-chain chlorinated paraffins in marine organisms from the Pearl River Estuary, South China. *Sci. Total Environ.* 671, 262–269 (2019).
8. Ma, X. et al. Air-seawater gas exchange and dry deposition of chlorinated paraffins in a typical inner sea (Liaodong Bay), North China. *Environ. Sci. Technol.* 52, 7729-7735 (2018).
9. Hu. H. et al. Spatial distribution, partitioning, and ecological risk of short chain chlorinated paraffins in seawater and sediment from East China Sea. *Sci. Total Environ.* 811, 151932 (2022).
10. Pan, X., Zhen, X., Tian, C. & Tang, J. Distributions, transports and fates of short- and medium-chain chlorinated paraffins in a typical river-estuary system. *Sci. Total Environ.* 751, 141769 (2021).
11. Gobas, F. A. P. C. A model for predicting the bioaccumulation of hydrophobic organic chemicals in aquatic food-webs: application to Lake Ontario. *Ecol. Model.* 69, 1-17 (1993).
12. Gobas, F. A. P. C., Zhang, X. & Wells, R. Gastrointestinal magnification: the mechanism of biomagnification and food chain accumulation of organic chemicals. *Environ. Sci. Technol.* 27, 2855–2863 (1993).
13. Wania, F. & Mackay, D. A global distribution model for persistent organic chemicals. *Sci. Total Environ.* 160, 211-232 (1995).
14. Gewurtz, S. B. et al. A comparison of contaminant dynamics in arctic and temperate fish: A modeling approach. *Chemosphere* 63, 1328-1341 (2006).
15. Chen, C. L., Löfstrand, K., Adolfsson-Erici, M., McLachlan, M. S. & MacLeod, M. Deriving in Vivo Bioconcentration Factors of a Mixture of Fragrance Ingredients Using a Single Dietary Exposure and Internal Benchmarking. *Environ. Sci. Technol.* 52, 5227–5235 (2018).
16. Arnot, J. A. & Gobas, F. A. A food web bioaccumulation model for organic chemicals in aquatic ecosystems. *Environ. Toxicol. Chem.* 23, 2343-2355 (2004).
17. Arnot, J. A. & Gobas, F. A. A review of bioconcentration factor (BCF) and bioaccumulation factor (BAF) assessments for organic chemicals in aquatic organisms. *Environ. Rev.* 14, 257-297 (2006).
18. Stewart, D. J., Weininger, D., Rottiers, D. V. & Edsall. T. An energetics model for lake trout, Salvelinus namaycush: application to the Lake Michigan population. *Can. J. Fish. Aquat. Sci.* 40, 681-698 (1993).
19. Barber, M. C., Suarez, L. A. & Lassiter, R. R. Modeling bioaccumulation of organic pollutants in fish with an application to PCBs in Lake Ontario salmonids. *Can. J. Fish. Aquat. Sci.* 48, 318-337 (1991).
20. Weininger, D. Accumulation of PCBs by lake trout in Lake Michigan. PhD dissertation, University of Wisconsin, Madison (1978).
21. Dong, S., Li, X., Su, X. & Wang, P. Concentrations and congener group profiles of short- and medium-chain chlorinated paraffins in animal feed materials. *Sci. Total Environ.* 647, 676–681 (2019).
22. Huang, Y. et al. Short-chain chlorinated paraffins in fish from two developed regions of China: Occurrence, influencing factors and implication for human exposure via consumption. *Chemosphere* 236, 124317 (2019).
23. Carubelli. G. et al. PCB contamination in farmed and wild sea bass (Dicentrarchus labrax L.) from a coastal wetland area in central Italy. *Chemosphere* 68, 1630-1635 (2007).
24. Malisch, R. & Kotz, A. Dioxins and PCBs in feed and food-review from European perspective. *Sci. Total Environ.* 491, 2-10 (2014).
25. Sijm, D., Seinen, W. & Opperhuizen. A. Life-cycle biomagnification study in fish. *Environ. Sci. Technol.* 26, 2162-2174 (1992).
26. Leontief, W. & Strout, A. *Multi-regional input-output analysis* (Macmillan, London, 1963).
27. Okamoto, N., Zhang, Y. & Zhao, K. Non-Survey Methods for Estimating Regional and Interregional Input-Output Multipliers. In: Okamoto, N., Ihara, T. (eds) Spatial Structure and Regional Development in China. IDE-JETRO Series. Palgrave Macmillan, London. <https://doi.org/10.1057/9780230524163_3> (2005).
28. Liu, W., Li, X., Liu, H., Tang, Z. & Guan, D. Estimating inter-regional trade flows in China: A sector-specific statistical model. *J. Geogr. Sci.* 25, 1247–1263 (2015).
29. Ihara, T. *Regional Economic Analysis* (Chiki no Keizai Bunseki, 1996).
30. Mi, Z. et al. A multi-regional input-output table mapping China's economic outputs and interdependencies in 2012. *Sci. Data* 5, 180155 (2018).
31. Liu, W., Li, X., Liu, H., Tang, Z. & Guan, D. Estimating inter-regional trade flows in China: A sector-specific statistical model. *J. Geogr. Sci.* 25, 1247–1263 (2015).
32. Bonfiglio, A. & Chelli, F. Assessing the behaviour of non-survey methods for constructing regional input–output tables through a Monte Carlo simulation. *Econ. Syst. Res.* 20, 243–258 (2008)
33. Natale, F., Borrello, A. & Motova, A. Analysis of the determinants of international seafood trade using a gravity model. *Mar. Policy* 60, 98−106 (2015).
34. Feng, K. et al. Outsourcing CO_2_ within China. *Proc. Natl. Acad. Sci. U.S.A.* 110, 11654-11659 (2013).
35. Chung, M. G., Kapsar, K., Frank, K. A. & Liu, J. The spatial and temporal dynamics of global meat trade networks. *Sci. Rep.* 10, 16657 (2020).
36. Liu, M. et al. Impacts of farmed fish consumption and food trade on methylmercury exposure in China. *Environ. Int.* 120, 333-344 (2018).
37. Deng, C. et al. Mercury risk assessment combining internal and external exposure methods for a population living near a municipal solid waste incinerator. *Environ. Pollut.* 219, 1060–1068 (2016).
38. Shang, X. H., Li, X. W., Zhang, L., Zhao, Y. F. & Wu, Y. N. Estimation of methylmercury intake from the 2007 Chinese Total Diet Study. *Food Addit. Contam. Part B Surveill* 3, 236–245 (2010).
39. Jiang, Q. T. et al. Human health risk assessment of organochlorines associated with fish consumption in a coastal city in China. *Environ. Pollut.* 136, 155–165 (2005).
40. Li, P. et al. Mercury in the seafood and human exposure in coastal area of Guangdong province, South China. Environ. *Toxicol. Chem.* 32, 541–547 (2013).
41. Qian, H. et al. Evaluating drivers and flow patterns of inter-provincial grain virtual water trade in China. *Sci. Total Environ.* 732, 139251 (2020).
42. Chungchunlam, S. M. S., Moughan, P. J., Garrick, D. P. & Drewnowski, A. Animal-sourced foods are required for minimum-cost nutritionally adequate food patterns for the United States. *Nat Food.* 1, 376-381 (2020).
43. Geyik, Ö., Hadjikakou, M. & Bryan, B. A. Climate-friendly and nutrition-sensitive interventions can close the global dietary nutrient gap while reducing GHG emissions. *Nat. Food.* 4, 61-73 (2023).
44. Gephart, J. A. et al. The environmental cost of subsistence: optimizing diets to minimize footprints. *Sci. Total Environ.* 553, 120–127 (2016).
45. Van-Dooren, C. A review of the use of linear programming to optimize diets, nutritiously, economically and environmentally. *Front. Nutr.* 5, 48 (2018).
46. Buttriss, J. L. et al. Diet modelling: how it can inform the development of dietary recommendations and public health policy. *Nutr. Bull.* 39, 115–125 (2014).
47. Zhuo, L. et al. Water for maize for pigs for pork: An analysis of inter-provincial trade in China. *Water Res.* 166, 115074 (2019).
48. Gao, S., Guo, G. & Dai, X. Research of inter-provincial grain transport costs based on GIS network analyst. *Logistics Sci-Tech.* 5, 135-139 (2014).
49. Yan, Y. R., Chen, J. L., Hou, G. & Jin, X. S. Feeding habits of Trichiurus lepturus in Beibu Gulf of South China Sea (in Chinese). *Chinese Journal of Applied Ecology* 21, 749-755 (2010).
50. He, X. B. Spatial distribution, Population Structure and Trophic Ecology of Common Trichiuridae Species in the Coastal Waters of China (in Chinese). Dissertation, JiMei University, Xiamen (2019).
51. Lin, L., Zhang, H., Li, H. & Cheng, J. Study on seasonal variation of the feeding habits of hairtail (Trichiurus japonicus) in the East China Sea (in Chinese). *Periodical of Ocean University of China* 36, 932-936 (2006).
52. Xu, J. Y., Chen, J. J., Tian, F. G. & Xu, Z. L. Summer diet composition and feeding ecology of large yellow croaker (Larimichthys crocea) in Guanjing Yang (in Chinese). *Journal of Fishery Sciences of China* 19, 94-104 (2012).
53. Yang, F. et al. Study on the Feeding ecology of Larimichthys crocea in the Ma’an archipelago based on biomarker and stomach content analysis (in Chinese). *Journal of Fishery Sciences of China* 30, 247-258 (2023).
54. Wei, X. J., Zhang, B., Shan, X. J., Jin, X. S. & Ren, Y. P. Feeding habits of small yellow croaker (Larimichthys polyactis) in the Bohai Sea (in Chinese). *Journal of Fishery Sciences of China* 25, 1289-1298 (2018).
55. Wang, K., Zhang, S. Y., Wang, Z. H., Xu, M. & Zhao, J. Feeding habits of small yellow croaker Larimichthys polyactis off Ma’an Archipelago. *Acta Hydrobiologica Sinica* 36, 1188-1192 (2012).
56. Song, Y. H., Xue Y., Xu, B. D., Zhang, C. L. & Ren, Y. P. Composition of food and niche overlap of three Sciaenidae species in Haizhou Bay (in Chinese). *Journal of Fishery Sciences of China* 44, 2017-2027 (2020).
57. Zhang, B. Feeding ecology of fishes in the Bohai Sea. *Prog. Fish. Sci.* 39, 11–22 (2018).
58. Fuji, T., Kasai, A., Suzuki, K. W., Ueno, M. & Yamashita, Y. Freshwater migration and feeding habits of juvenile temperate seabass Lateolabrax japonicus in the stratified Yura River estuary, the Sea of Japan. Fish Sci. 2010, 76, 643–652.
59. Li, J. Food composition and feeding habits of Japanese sea bass in the Bohai Sea. *Mar. Sci.* 6, 39-44 (1994).
60. Watanabe, H., Kubodera, T. & Kawahara, S. Feeding habits of Pacific pomfret Brama japonica in the transition zone of the central North Pacific. *Fish. Sci.* 69, 269–276 (2003).
61. Watanabe, H., Kubodera, T. & Kawahara, S. Summer feeding habits of the pacific pomfret brama japonica in the transitional and subarctic waters of the central north pacific. *J. Fish Biol.* 68, 1436-1450 (2006).
62. Drouillard, K. G., Tomy, G. T., Muir, D. C. G. & Friesen, Ken. J. Volatility of chlorinated n‐alkanes (C_10_−C_12_): Vapor pressures and Henry's law constants. *Environ. Toxicol. Chem.* 17, 1252−1260 (1998).
63. European Chemicals Bureau. European Union Risk Assessment Report: Alkanes, C10-13, Chloro- (European Chemical Bureau, Luxembourg, 2000).
64. European Chemicals Bureau. Updated Risk Assessment of Alkanes, C10-13, Chloro- (European Chemical Bureau, Luxembourg, 2008).
65. Krogseth, I. S. et al. Evaluating the environmental fate of short-chain chlorinated paraffins (SCCPs) in the Nordic environment using a dynamic multimedia model. *Environ. Sci. Processes Impacts* 15, 2240−2251 (2013).
66. MacLeod, M., Fraser, A. J. & Mackay, D. Evaluating and expressing the propagation of uncertainty in chemical fate and bioaccumulation models. *Environ. Toxicol. Chem.* 21, 700−709 (2002).
67. Ma, X. et al. Congener-specific distribution and bioaccumulation of short-chain chlorinated paraffins in sediments and bivalves of the Bohai Sea, China. *Mar. Pollut. Bull.* 79, 299-304 (2014).
68. Zhang, Q. Q. et al. Multimedia fate modeling and risk assessment of a commonly used azole fungicide climbazole at the river basin scale in China. *Sci. Total Environ.* 520, 39-48 (2015).
69. Ma, X. et al. Bioaccumulation and trophic transfer of short chain chlorinated paraffins in a marine food web from Liaodong Bay, North China. *Environ. Sci. Technol.* 48, 5964-5971 (2014).
70. Nishigaki, A., Muramatsu, A., Onozato, M. & Ohshima, S. Determination of polycyclic aromatic hydrocarbons in the fecal materials of Mactra veneriformis and excrement of Marphysa sanguinea. *Polycycl. Aromat. Comp.* 33, 151-160 (2013).
71. Yuan, B. et al. Short chain chlorinated paraffins in mollusks from coastal waters in the Chinese Bohai Sea. *Environ. Sci. Technol.* 46, 6489-6496 (2012).
72. Jiang, W. et al. Contamination of short-chain chlorinated paraffins to the biotic and abiotic environments in the Bohai Sea. *Environ. Pollut.* 233, 114-124 (2018).
73. United Naitons Environment Programme. Report of the Persistent Organic Pollutants Review Committee on the work of its eleventh meeting. UNEP/POPS/POPRC. 11/10/Add.2. (Persistent Organic Pollutants Review Committee, Geneva, 2015).
74. Bekele, T. G., Zhao, H., Wang, Q. & Chen, J. Bioaccumulation and trophic transfer of emerging organophosphate flame retardants in the marine food webs of Laizhou Bay, North China. *Environ. Sci. Technol.* 53, 13417-13426 (2019).
75. Wang, S. et al. Bioaccumulation of organophosphorus flame retardants in marine organisms in Liaodong Bay and their potential ecological risks based on species sensitivity distribution. *Environ. Pollut.* 317, 120812 (2023).
76. Storelli, M. M. Potential human health risks from metals (Hg, Cd, and Pb) and polychlorinated biphenyls (PCBs) via seafood consumption: estimation of target hazard quotients (THQs) and toxic equivalents (TEQs). *Food Chem. Toxicol.* 46, 2782–2788 (2008).
77. Perugini, M. et al. Levels of polychlorinated biphenyls and organochlorine pesticides in some edible marine organisms from the central Adriatic Sea. *Chemosphere* 57, 391–400 (2004).
78. Çakıroğulları, G. Ç., Uçar, Y., Oymael, B., Bozkurt, E. N. & Kılıc, D. PCDD/F, dl-PCB and indicator PCBs in whiting, horse mackerel and anchovy in Black Sea in Turkey. *Turk. J. Fish. Aquat. Sci.* 10, 357–362 (2010).
79. Miniero, R. et al. Persistent toxic substances in Mediterranean aquatic species. *Sci. Total Environ.* 494, 18-27 (2014).
80. Litz, M. N. et al. Effects of variable oceanographic conditions on forage fish lipid content and fatty acid composition in the northern California Current. *Mar. Eco. Prog. Ser.* 405, 71–85 (2010).
81. Bayarri, S., Baldassarri, L. T., Iacovella, N., Ferrara, F. & di-Domenico, A. PCDDs, PCDFs, PCBs and DDE in edible marine species from the Adriatic Sea. *Chemosphere* 43, 601–610 (2001).
82. Nakagawa, R. et al. Hexabromocyclododecane determination in seafood samples collected from Japanese coastal areas. *Chemosphere* 81, 445-452 (2010).
83. Tanaka, H., Kono, K. & Ueno, D. Bioaccumulation of Dioxins in the Benthic Fish from Hiroshima Bay. *Interdisciplinary Studies on Environmental Chemistry-Environmental Research in Asia* 47-54 (2009).
84. Byun, G. H., Moon, H. B., Choi, J. H., Hwang, J. & Kang, C. K. Biomagnification of persistent chlorinated and brominated contaminants in food web components of the Yellow Sea. *Mar. Pollut. Bull.* 73, 210-219 (2013).
85. Zhuang, H., Liu, J., Zhong, Y., Cui, L. & Luo, H. Analysis of fatty acids components in muscles of eight species of cardinalfishes (Apogonidae). *Food and Fermentation Industries* 46, 266-271 (2020).
86. Li, G., Sinclair, A. J. & Li, D. Comparison of lipid content and fatty Acid composition in the edible meat of wild and cultured freshwater and marine fish and shrimps from China. *J. Agric. Food Chem.* 59, 1871-1881 (2011).
87. Hiroaki, S. & Kenji, I. Docosahexaenoic acid content of fatty acids in the lipids of two species of frigate mackerel, Auxis rocheri and Auxis thazard. *Biosci. Biotechnol. Biochem.* 60, 1014-1016 (1996).
88. Lenky, C., Eisert, R., Oftedal, O. T. & Metcalf, V. Proximate composition and energy density of nototheniid and myctophid fish in McMurdo Sound and the Ross Sea, Antarctica. *Polar. Biol.* 35, 717–724 (2012).
89. Stowasser, G., Pond, D. W. & Collins, M. A. Using fatty acid analysis to elucidate the feeding habits of Southern Ocean mesopelagic fish. *Mar. Biol.* 156, 2289–2302 (2009).
90. Parzanini, C., Parrish, C. C., Hamel, J. F. & Mercier, A. Functional diversity and nutritional content in a deep-sea faunal assemblage through total lipid, lipid class, and fatty acid analyses. *PLoS One* 13, e0207395 (2018).
91. Koizumi, K., Hiratsuka, S. & Saito, H. Lipid and fatty acids of three edible myctophids, Diaphus watasei, Diaphus suborbitalis, and Benthosema pterotum: high levels of icosapentaenoic and docosahexaenoic acids. *J. Oleo. Sci.* 63, 461-470 (2014).
92. Wan, Y. et al. Characterization of trophic transfer for polychlorinated dibenzo-p-dioxins, dibenzofurans, non- and mono-ortho polychlorinated biphenyls in the marine food web of Bohai Bay, North China. *Environ. Sci. Technol.* 39, 2417-2425 (2005).
93. Wan, Y., Jin, X., Hu, J. & Jin, F. Trophic dilution of polycyclic aromatic hydrocarbons (PAHs) in a marine food web from Bohai Bay, north China. *Environ. Sci. Technol.* 41, 3109-3114 (2007).
94. Wu, L., Tang, W. Q. & Zhang, Y. Research on the differences of anadromous migratory distance between Coilia mystus and Coilia nasus based on the transfer process of body lipid. *Journal of Fisheries of China* 41, 212-220 (2017).
95. Zhang, X. et al. Fatty acid composition analyses of commercially important fish species from the Pearl River Estuary, China. *PLoS One* 15, e0228276 (2020).
96. Su, G. Y. et al. Polybrominated diphenyl ethers and their methoxylated metabolites in anchovy (Coilia sp.) from the Yangtze River Delta, China. *Environ. Sci. Pollut. Res. Int.* 17, 634–642 (2010).
97. Sun, R. et al. Short-chain chlorinated paraffins in marine organisms from the Pearl River Estuary in South China: Residue levels and interspecies differences. *Sci. Total Environ.* 553, 196-203 (2016).
98. Liu, Y. et al. Brominated flame retardants (BFRs) in marine food webs from Bohai Sea, China. *Sci. Total Environ.* 772, 145036 (2021).
99. Shang, X. et al. Polybrominated diphenyl ethers (PBDEs) and indicator polychlorinated biphenyls (PCBs) in various marine fish from Zhoushan fishery, China. *Food Control* 67, 240–246 (2016).
100. Zhang, C. et al. Polycyclic aromatic hydrocarbons (PAHs) in marine organisms from two fishing grounds, South Yellow Sea, China: Bioaccumulation and human health risk assessment. *Mar. Pollut. Bull.* 153, 110995 (2020).
101. Zheng, H. et al. Study of Spinyhead Croaker (Collichthys lucidus) Fat Content Forecasting Model Based on Electronic Nose and Non-linear Data Resolution Model. *Food Anal. Methods* 12, 1927–1937 (2019).
102. Huang, Q. et al. Bioaccumulation and Trophic Transfer of Organophosphate Flame Retardants and Their Metabolites in the Estuarine Food Web of the Pearl River, China. *Environ. Sci. Technol.* 57, 3549-3561 (2023).
103. Yu, Z. et al. Bioaccumulation of polycyclic aromatic hydrocarbons (PAHs) in wild marine fish from the coastal waters of the northern South China Sea: Risk assessment for human health. *Ecotoxicol. Environ. Saf.* 180, 742–748 (2019).
104. Artharn, A., Benjakul, S., Prodpran, T. & Tanaka, M. Properties of a protein-based film from round scad (Decapterus maruadsi) as affected by muscle types and washing. *Food Chem.* 103, 867–874 (2007).
105. Bockus, A. B. & Seibel, B. A. Trimethylamine oxide accumulation as a function of depth in Hawaiian mid-water fishes. *Deep Sea Res. Pt I.* 112, 37–44 (2016).
106. Hayashi, K. & Takagi, T. Seasonal variations in lipids and fatty acids of Japanese anchovy, Engraulis japonica. *Bull. Fac. Fish Hokkaido. Univ.* 29, 38–47 (1978).
107. Selmi, S. & Sadok, S. The influence of season on lipid content and fatty acids profile of Euthynnus alletteratus fillets and by-products. *J. Muscle Foods* 21, 365-378 (2010).
108. Sun, R. et al. Polycyclic aromatic hydrocarbons in surface sediments and marine organisms from the Daya Bay, South China. *Mar. Pollut. Bull.* 103, 325-332 (2016).
109. Özdemir, N. Ş., Parrish, C. C., Parzanini, C. & Mercier, A. Neutral and polar lipid fatty acids in five families of demersal and pelagic fish from the deep Northwest Atlantic. *ICES J. Mar. Sci.* 76, 1807–1815 (2019).
110. Takahashi, S., Tanabe, S. & Kawaguchi, K. Organochlorine and Butyltin Residues in Mesopelagic Myctophid Fishes from the Western North Pacific. *Environ. Sci. Technol.* 34, 5129–5136 (2000).
111. Jiang, G. et al. Contamination characteristics of short-chain chlorinated paraffins in edible fish of Shanghai. *Environ. Sci.* 34, 3374-3380 (2013).
112. Wan, Y., Hu, J., Zhang, K. & An, L. Trophodynamics of polybrominated diphenyl ethers in the marine food web of Bohai Bay, North China. *Environ. Sci. Technol.* 42, 1078-1083 (2008).
113. Mathew, S., Ammu, K., Viswanathan Nair, P. G. & Devadasan, K. Cholesterol content of Indian fish and shellfish. *Food Chem.* 66, 455–461 (1999).
114. Devadason, C. et al. Comparative analysis of lipid content and fatty acid composition of commercially important fish and shellfish from Sri Lanka and Japan. *J. Oleo. Sci.* 65, 543-556 (2016).
115. Rahayu, S. M., Suseno, S. H. & Ibrahim, B. Proximate, Fatty Acid Profile and Heavy Metal Content of Selected By-Catch Fish Species from Muara Angke, Indonesia. *Pak. J. Nutr.* 13, 480-485 (2014).
116. Lee, D. S. et al. Biochemical Composition of Muscle from Tanaka’s Eelpout Lycodes tanakae, Magistrate Armhook Squid Berryteuthis magister, and Ocean Sunfish Mola mola, Caught in the East Sea, Korea. *Fish. Aquatic Sci.* 15, 99-105 (2012).
117. López-Huerta, J. M., Vega-Villasante, F., Viana, M. T., Carrillo-Farnés, O. & Badillo-Zapata, D. First report of nutritional quality of the native fish Dormitator latifrons (Richardson, 1844) (Perciformes: Eleotridae). *Lat. Am. J. Aquat. Res.* 46 (2018).
118. Goda, A., El-Husseiny, O., Abdul-Aziz, G., Suloma, A. & Ogata, Y. Fatty acid and free amino acid composition of muscles and gonads from wild and captive tilapia oreochromis niloticus (L.) (Teleostei: Perciformes): An approach to development broodstock diets. *J. Fish. Aquatic Sci.* 2, 86-99 (2007).
119. Mohanty, B. P. et al. Nutritional composition of food fishes and their importance in providing food and nutritional security. *Food Chem.* 293, 561-570 (2019).
120. Spiros, Z. & Kostas, L. Seasonal variation in the fatty acid composition of three Mediterranean fish – sardine (Sardina pilchardus), anchovy (Engraulis encrasicholus) and picarel (Spicara smaris). *Food Chem.* 103, 725–728 (2007).
121. Spitz, J., Mourocq, E., Schoen, V. & Ridoux, V. Proximate composition and energy content of forage species from the Bay of Biscay: high- or low-quality food? *ICES J. Mar. Sci.* 67, 909–915 (2010).
122. Edirisinghe, E., Perera, W. & Bamunuarachchi, A. Nutritional evaluation of some small coastal fish in Sri Lanka. *J. Natl. Aquat. Resour. Res. Dev. Agency* 36, 47-53 (2000).
123. Siriskar, D. A., Khedkar, G. D. & Lior, D. Production of salted and pressed anchovies (stolephorus sp.) and it’s quality evaluation during storage. *J. Food Sci. Technol.* 50, 1172–1178 (2013).
124. Senarath, S. et al. Quantitative Analysis of the Distribution of cis-Eicosenoic Acid Positional Isomers in Marine Fishes from the Indian Ocean. *J. Oleo. Sci.* 66, 187-197 (2017).
125. Donnelly, J., Stickney, D. G. & Torres, J. J. Proximate and elemental composition and energy content of mesopelagic crustaceans from the Eastern Gulf of Mexico. *Mar. Biol.* 115, 469–480 (1993).
126. Liu, J. et al. Decrease of lipid oxidation for dried shrimp (Acetes chinensis) preservation using alkaline lipase hydrolysis technology. *J. Aquat. Food Prod. T.* 25, 169–176 (2016).
127. Li, X. et al. Nutritional evaluation, flavor characteristics and microbial community of shrimp paste made from different materials and variance analysis. *Food Chemistry Advances* 2, 100268 (2023).
128. Tou, J. C., Jaczynski, J. & Chen, Y. C. Krill for human consumption: nutritional value and potential health benefits. *Nutr. Rev.* 65, 63–77 (2007).
129. Lokman, S. Food value of indigenous zooplankton from the South China Sea on the east coast of Peninsular Malaysia. *J. World Aquacult. Soc.* 25, 208–213 (1994).
130. Yamada, H. et al. Lipids, fatty acids and hydroxy-fatty acids of Euphausia pacifica. *Sci. Rep.* 7, 9944 (2017).
131. Saito, H. et al. High levels of n-3 polyunsaturated fatty acids in Euphausia pacifica and its role as a source of docosahexaenoic and icosapentaenoic acids for higher trophic levels. *Mar. Chem.* 78, 9–28 (2002).
132. Yoshitomi, B. & Yamaguchi, H. Chemical composition of dried eyeballs from Euphausia superba and Euphausia pacifica. *Fish Sci.* 73, 1186–1194 (2007).
133. Parrish, C. C., Deibel, D. & Thompson, R. J. Effect of sinking spring phytoplankton blooms on lipid content and composition in suprabenthic and benthic invertebrates in a cold ocean coastal environment. *Mar. Ecol. Prog. Ser.* 391, 33–51 (2009).
134. Mayzaud, P., Virtue, P. & Albessard, E. Seasonal variations in the lipid and fatty acid composition of the euphausiid Meganyctiphanes norvegica from the Ligurian Sea. *Mar. Ecol. Prog. Ser.* 186, 199–210 (1999).
135. Adare, K. I. & Lasenby, D. C. Seasonal changes in the total lipid content of the opossum shrimp, Mysis relicta (Malacostraca: Mysidacea). *Can. J. Fish. Aquat. Sci.* 51, 1935–1941 (1994).
136. Goswami, S. C., Rao, T. S. S. & Matondkar, S. G. P. Biochemical composition of zooplankton from the Andaman Sea. *Indian J. Mar. Sci.* 10, 296–300 (1981).
137. Childress, J. J. & Price, M. H. Growth rate of the bathypelagic crustacean Gnathophausia ingens (Mysidacea: Lophogastridae). *Mar. Biol.* 76, 165-177 (1983).
138. Shao, M., Tao, P., Wang, M., Jia, H. & Li, Y. F. Trophic magnification of polybrominated diphenyl ethers in the marine food web from coastal area of Bohai Bay, North China. *Environ. Pollut.* 213, 379-385 (2016).
139. Yongquan, S., Hong, Y. & Huan, Z. Contents of carbohydrates and lipids in the bodies of two planktonic crustaceans. *Chin. J. Ocean. Limnol.* 8, 46–49 (1990).
140. Wan, L., Peng, Y. Y., Yu, H. H., Xu, W. J. & He, J. Comparing the Muscle Nutritional Quality of Eight Common Wild-Caught Economic Shrimp Species from the East China Sea. *J. Aquat. Food Prod. Technol.* 31, 549–564 (2022).
141. Gorbatenko, K. M. & Nadtochy, V. A. The biochemical composition and caloric content of the macrozoobenthos of the western Kamchatka shelf. *Russ. J. Mar. Biol*. 44, 283–291 (2018).
142. Wang, M. & Jeffs, A. G. Nutritional composition of potential zooplankton prey of spiny lobster larvae: a review. *Rev. Aquacult.* 6, 270-299 (2014).
143. Figueiredo, J., Penha-Lopes, G., Anto, J., Narciso, L. & Lin, J. Potential fertility and egg development (volume, water, lipid, and fatty acid content) through embryogenesis of uca rapax (decapoda: brachyura: ocypodidae). *J. Crustacean Biol.* 28, 528–533 (2008).
144. Childress, J. J. & Nygaard, M. Chemical composition and buoyancy of midwater crustaceans as function of depth of occurrence off Southern California. *Mar. Biol.* 27, 225–238 (1974).
145. Herawati, V. E. et al. The effect of probiotic bacteria in culture media using organic fertilizer for population density, biomass production and nutrient quality of Phronima sp. as natural feed. *Aquac. Res.* 51, 836–843 (2020).
146. Stevens, C. J. et al. Total lipid and fatty acid composition of mesozooplankton functional group members in the NE Pacific over a range of productivity regimes. *Mar. Ecol. Prog. Ser.* 687, 43-64 (2022).
147. Sun, H. et al. Characterization of lipid composition in the muscle tissue of four shrimp species commonly consumed in China by UPLC-Triple TOF-MS/MS. *LWT* 128, 109469 (2020).
148. Schukat, A., Auel, H., Teuber, L., Lahajnar, N. & Hagen, W. Complex trophic interactions of calanoid copepods in the Benguela upwelling system. *J. Sea Res.* 85, 186–196 (2014).
149. Nagabhushanam, R. & Lomte, V. S. Biochemical studies in the freshwater mussel, Parreysia corrugata. *Hydrobiologia* 37, 545–552 (1971).
150. Yang, C. et al. Characterization and distribution of polybrominated diphenyl ethers in shellfish in Shenzhen coastal waters and assessment of human health risks. *Mar. Pollut. Bull.* 191, 114957 (2023).
151. Hayashi, K. & Kishimura, H. Amount and composition of diacyl glyceryl ethers in various tissue lipids of the deep-sea squid berryteuthis magister. *J. Oleo Sci.* 51, 523-529 (2002).
152. Takaaki, S. et al. Taste Components of Boreo Pacific Gonate Squid Gonatopsis borealis. *Fish. Sci.* 63, 772-778 (1997).
153. Sinclair, E. H., Walker, W. A. & Thomason, J. R. Body Size Regression Formulae, Proximate Composition and Energy Density of Eastern Bering Sea Mesopelagic Fish and Squid. *PLoS One* 10, e0132289 (2015).
154. Neifar, A., Ben-Rebah, F., Gargouri, A. & Abdelmouleh, A. Physicochemical characterization of Sepia officinalis ink and the effects of storage conditions on the coagulation process. *J. Mar. Biolog.* 89, 803-807 (2009).
155. Soliman, Y. S. & Wade, T. L. Estimates of PAHs burdens in a population of ampeliscid amphipods at the head of the Mississippi Canyon (N. Gulf of Mexico). *Deep Sea Res. Part II Top. Stud. Oceanogr.* 55, 2577-2584 (2008).
156. Baeza-Rojano, E., Hachero-Cruzado, I. & Guerra-García, J. M. Nutritional analysis of freshwater and marine amphipods from the Strait of Gibraltar and potential aquaculture applications. *J. Sea Res.* 85, 29–36 (2014).
157. Lease, H. M. & Wolf, B. O. Lipid content of terrestrial arthropods in relation to body size, phylogeny, ontogeny and sex. *Physiol. Entomol.* 36, 29-38 (2011).
158. Pethybridge, H. R., Nichols, P. D., Virtue, P. & Jackson, G. D. The foraging ecology of an oceanic squid, Todarodes filippovae: The use of signature lipid profiling to monitor. *Deep Sea Res. Part II* 95, 119-128 (2013).
159. Prato, E., Danieli, A., Maffia, M. & Biandolino, F. Lipid Contents and Fatty Acid Compositions of Idotea baltica and Sphaeroma serratum (Crustacea: Isopoda) as Indicators of Food Sources. *Zool. Stud.* 51, 38-50 (2012).
160. Lin, S. G., Wang, S. K. & Li, J, S. The feeding experimemt of larvae Pseudosciaena crocea with formula feeds. *Journal of Fujian Agriculture and Forestry University* 1, 92-93 (2002).
161. Liu, C., Zhang, Y. Z. & Ma, L. SWOT analysis of feeding rhubarb with feed in China (in Chinese). *China Fisheries* 12, 57-61 (2021).
162. Liu, Z. K. Present research condition of Larimichthys crocea feed in Ningde of Fujian province (in Chinese). *Journal of Ningde Normal University* 26, 229-234 (2014).
